# New Alkaloids From a Hawaiian Fungal Strain *Aspergillus felis* FM324

**DOI:** 10.3389/fchem.2021.724617

**Published:** 2021-08-09

**Authors:** Cong Wang, Ariel M. Sarotti, KH Ahammad Uz Zaman, Xiaohua Wu, Shugeng Cao

**Affiliations:** ^1^ Department of Pharmaceutical Sciences, Daniel K. Inouye College of Pharmacy, University of Hawai’i at Hilo, Hilo, HI, United States; ^2^ Key Laboratory of Chemistry and Engineering of Forest Products, State Ethnic Affairs Commission, Guangxi Key Laboratory of Chemistry and Engineering of Forest Products, Guangxi Collaborative Innovation Center for Chemistry and Engineering of Forest Products, School of Chemistry and Chemical Engineering, Guangxi University for Nationalities, Nanning, China; ^3^ Facultad de Ciencias Bioquímicas y Farmacéuticas, Instituto de Química Rosario (CONICET), Universidad Nacional de Rosario, Rosario, Argentina

**Keywords:** *Aspergillus felis*, trichocomaceae, alkaloids, antiproliferative, antibacterial, NF-κB inhibitory activities

## Abstract

Two new alkaloids tryptoquivaline Y (1) and pseurotin I (2), together with eight known compounds (3–10), were purified from a fungal strain *Aspergillus felis* FM324, which was isolated from a Hawaiian beach soil sample. The absolute configuration and physicochemical data of tryptoquivaline Z (3) were reported for the first time here in this paper. Compound 1 is an uncommon tryptoquivaline analog containing a 3-*O*-isobutanoyl group. The structures of the new compounds 1–2 and known compound 3 were elucidated through HRESIMS, NMR spectroscopy and ECD analysis. All the compounds were evaluated for their antiproliferative, antibacterial and NF-κB inhibitory activities. Compound 4 showed weak antibacterial activity against *Staphylococcus aureus*, methicillin resistant *Staphylococcus aureus* and *Bacillus subtilis* with the same MIC value of 59.2 µM. Compounds **3** and **2** inhibited NF-κB with IC_50_ values of 26.7 and 30.9 μM, respectively.

## Introduction

Marine fungi remain one of the few underexplored resources of natural products (Overy et al., 2019), and they have become the main source of new compounds from marine microorganisms due to their complex genetic background (Zhao et al., 2016). Most of the reported marine fungal secondary metabolites showed certain biological properties including antibacterial (Wang et al., 2021) and anticancer (Deshmukh et al., 2017) activities. *Aspergillus* is a huge and diverse fungal genus (Ibrahim and Asfour, 2018), ubiquitously found in soil, terrestrial plants, animals and marine. Totally, there are about 380 species in the genus *Aspergillus*. As a dominant and the most studied fungal genus in endophytes, more than 350 new fungal metabolites were isolated from *Aspergillus* during 2015–2019 (Vadlapudi et al., 2017). Marine *Aspergillus* sp. produced plenty of secondary metabolites including polyketides, sterols, fatty acids, peptides, alkaloids, terpenoids and miscellaneous compounds, which exhibited different pharmacological activities such as antimicrobial, cytotoxicity, anti-inflammatory and antioxidant activity (Ibrahim et al., 2017a; Ibrahim et al., 2017b; Elkhayat, et al., 2016; Mohamed, et al., 2020). In the past few years, our research group has studied the secondary metabolites of some marine fungi including different *Aspergillus* species from Hawaii. These secondary metabolites had different types of structures and exhibited various biological activities (Li et al., 2015; Fei-Zhang et al., 2016; Li et al., 2016; Huang et al., 2017; Li et al., 2018; Li et al., 2019; Wang et al., 2019; Zaman et al., 2020a; Wang et al., 2020; Zaman et al., 2021). In our continuing search for bioactive molecules from Hawaiian fungi, we studied an extract of *Aspergillus felis* FM324, which led to the separation and identification of ten compounds (1–10). Here, we report two new molecules (1–2) along with eight known secondary metabolites (3–10). Compounds 1–10 were evaluated for their NF-κB inhibitory property and anti-proliferative activity against A2780 as well as their antibacterial potential against both Gram-positive and Gram-negative bacteria.

## Materials and Methods

### General Experimental Procedures

Optical rotations, CD, UV and FT-IR spectra were measured with a Rudolph Research analytical AutoPol automatic polarimeter (Rudolph Research Analytical, NJ, United States), JASCO J-815 CD (Jasco Corporation, Japan), Shimadzu UV spectrophotometer UV-1800 and Thermo Scientific Nicolet iS10 IR spectrometer (Thermo Fisher Scientific, WI, United States), respectively. The structure characterizations of all the compounds were based on 1D NMR (^1^H, ^13^C) and 2D NMR (COSY, HSQC, HMBC, 1D-NOE and ROESY) data, recorded on a Bruker AM-400 spectrometer (Bruker BioSpin AG, Switzerland). An Agilent 6,530 Accurate-Mass Q-TOF LC-MS spectrometer (Agilent Technologies, Germany) was used to record high-resolution mass spectra. Preparative RP-HPLC was carried out on an Ultimate 3000 chromatographic system (Agilent Technologies, Germany) with a Phenomenex preparative column (Phenyl-hexyl, 5 μ, 100 × 21.2 mm) and semipreparative RP-HPLC on an Ultimate 3000 chromatographic system (Agilent Technologies, Germany) with a Phenomenex semipreparative column (C_18_, 5 μ, 250 × 10 mm), connected to a Dionex Ultimate 3000 DAD detector (Agilent Technologies, Germany) (detected at 210, 254, 320, and 365 nm) and a Dionex Ultimate 3000 automated fraction collector. All solvents were HPLC grade. Diaion HP-20 (Alfa Aesar, Japan) was used to run the open-column chromatography.

### Strain Isolation and Fermentation

The strain FM324 was isolated from a sample collected at a beach at Kona, the Big Island, Hawaii. The strain was deposited in an −80°C freezer at Daniel K. Inouye College of Pharmacy, University of Hawaii at Hilo, HI, United States. The strain was grown on PDA plates at 28°C for 3 days, then it was cut into small pieces and inoculated into an autoclaved liquid PDB medium (20 L) for fermentation at 24°C for 30 days.

### Molecular Identification of the Fungal Strain M324

DNA extraction: DNA was extracted according to the literature (Liu et al., 2000), with slight modifications. Mycelium was added to 500 µl of lysis buffer (400 mM Tris-HCl [pH 8.0], 60 mM EDTA, 150 mM NaCl, 1% sodium dodecyl sulfate) and incubated at 85°C for 20 min. After adding 150 µl of 3 M sodium acetate (pH 5.2), the tube was vortexed briefly and centrifuged (12,500rpm) for 1 min. The supernatant was transferred to another tube and centrifuged again. After transferring the supernatant to a new tube, an equal volume of isopropanol was added and mixed by inversion. The tube was centrifuged for 2 min and the supernatant was discarded. The DNA pellet was washed twice with 300 µl of 70% ethanol. The DNA was air dried at room temperature for 45 min, then dissolved in 100 µL of 10 mM Tris-HCl (pH 8.0). Sequencing of ITS region: The ITS region was amplified with the ITS1 and ITS4 primers. The PCR reaction included 1X High Fidelity PCR Buffer (Invitrogen), 2 mM MgSO4, 0.2 mM dNTP mix, 4% DMSO, 0.2 µM of each primer, 1 U Platinum Taq DNA Polymerase High Fidelity (Invitrogen), and 10 ng of genomic DNA. The PCR cycling conditions were 95°C for 3 min, followed by 35 cycles of 95°C for 30 s, 50°C for 30 s and 72°C for 1 min, and a final extension of 72°C for 5 min. The PCR product was purified using Mag-Bind Total Pure NGS beads (Omega Bio-tek), then sequenced using a 3730xl DNA Analyzer (Applied Biosystems). The sequence was compared to the NCBI nucleotide collection (limited to sequences from type material) using the Basic Local Alignment Search Tool (BLAST), and was deposited in GenBank under the accession no. MZ227547.

### Extraction and Isolation

After filtration of the fermentation broth, the mycelia of FM324 were extracted three times with acetone. Acetone was removed by evaporation in vacuum. After combining the aqueous mycelia extraction and supernatant solution, it was subjected to HP-20 column eluted with MeOH-H_2_O into four fractions (30, 50, 90 and 100% MeOH). Fraction 3 (3.21 g) was separated by using prep-HPLC (Phenyl-Hexyl, 100 × 21.20 mm, 5 μm; 8 ml/min) eluted with 40–100% MeOH-H_2_O in 20 min to yield 26 sub-fractions (SFr3–1∼26). SFr 3–11 (180 mg) was purified by semi-preparative HPLC (38% MeCN/H_2_O, v/v, 1.0% formic acid, 3.0 ml/min) over a C18 column to afford compound **1** (1.6 mg, *t*_R_ 32.2 min). SFr 3–14 (152.7 mg) was purified by semi-preparative HPLC (20% MeCN/H_2_O, v/v, 1.0% formic acid, 3.0 ml/min) over a C18 column to afford compounds **2** (1.2 mg, *t*_R_ 35.3 min) and **7** (2.2 mg, *t*_R_ 21.4 min). SFr 3–9 (720 mg) was purified by semi-preparative HP2LC (30% MeCN/H_2_O, v/v, 1.0% formic acid, 3.0 ml/min) over a C18 column to afford compounds **3** (2.7 mg, *t*_R_ 27.6 min) and **5** (1.6 mg, *t*_R_ 8.3 min). SFr 3–20 (100.1 mg) was purified by semi-preparative HPLC (60% MeCN/H_2_O, v/v, 1.0% formic acid, 3.0 ml/min) over a C18 column to afford compound **4** (1.1 mg, *t*_R_ 16.5 min). SFr 3–17 (6.3 mg) afford compound **6** (6.3 mg). SFr 3–12 (201.8 mg) was purified by semi-preparative HPLC (30% MeCN/H_2_O, v/v, 1.0% formic acid, 3.0 ml/min) over a C18 column to afford compounds **8** (12.6 mg, *t*_R_ 19.8 min), **9** (5.2 mg, *t*_R_ 23.9 min) and **10** (1.0 mg, *t*_R_ 26.3 min).

Tryptoquivaline Y (**1**): White amorphous powder; ${\left[\text{α}\right]}_{D}^{25}$ +135 (*c* 0.10, MeOH); UV (MeOH) λ_max_ (log *ε*) 212 (4.42), 302 (3.47) nm; CD (0.10 mM, MeOH) λ_max_ (Δε) 224 (−29.85), 250 (+21.67), 288 (+16.88) nm; IR (KBr) *ν*_max_ 3,335, 2,921, 2,847, 1,651, 1,613, 1,519, 1,418, 1,375, 1,344, 1,271, 1,235, 1,083, 1,050, 1,033, 748.4 cm^−1^; ^1^H and ^13^C NMR data (see Table 1); HRESIMS *m/z*521.2042 [M + H]^+^ (calcd for C_27_H_29_N_4_O_7_, 521.2031).

**TABLE 1:** ^1^H (400 MHz) and ^13^C (100 MHz) NMR data of compounds 1–3 in DMSO-*d*_*6*_.

No	1		2		3	
	*δ*_C_	*δ*_H_ (*J* in Hz)	*δ*_C_	*δ*_H_ (*J* in Hz)	*δ*_C_	*δ*_H_ (*J* in Hz)
2	85.1, CH	5.08 (s)	187.0, C	-	84.7, CH	4.88 (s)
3	84.1, C	-	111.6, C	-	77.3, C	
4	139.8, C	-	196.8, C	-	138.2, C	
5	123.8, CH	7.36 (d, 6.5)	92.5, C	-	124.8, CH	7.38 (d, 7.8)
6	124.7, CH	7.09 (m)	166.6, C	-	124.7, CH	7.11 (dd, 7.8, 6.6)
7	130.0, CH	7.35 (overlap)	-	-	129.5, CH	7.11 (dd, 7.8, 6.6)
7-NH	-	-	-	9.95 (s)	-	-
8	114.9, CH	7.35 (overlap)	91.2, C	-	114.7, CH	7.42 (d, 7.8)
9	133.6, C	-	75.0, CH	4.40 (brs)	137.1, C	-
10	170.1, C		71.9, CH	4.33 (d, 5.8)	170.7, C	-
11	58.0, CH	5.29 (brs)	68.3, CH	4.45 (ddd, 5.8, 7.8, 11.0)	58.6, CH	5.22 (brs)
12	38.2, CH_2_	2.81 (d, 14.4)	129.9, CH	5.42 (overlap)	38.0, CH_2_	2.74 (d, 15.6)
		3.15 (dd,14.4, 7.7)				2.81 (dd,15.6, 7.0)
13	173.1, C	-	132.0, CH	5.44 (overlap)	172.3, C	-
14	69.4, C	-	29.3, CH_2_	2.02 (m); 1.97 (m)	69.6, C	-
15		-	22.2, CH_2_	1.31 (m); 1.28 (m)	-	-
16		-	13.6, CH_3_	0.82 (t, 7.3)	-	-
17	160.2, C	-	5.7, CH_3_	1.63 (s)	159.9, C	-
18	121.6, C	-	196.4, C	-	121.8, C	-
19	126.1, CH	8.19 (d, 7.0)	133.4, C	-	126.1, CH	8.18 (d, 8.0)
20	127.1, CH	7.59 (t, 7.0)	130.3, CH	8.25 (overlap)	127.0, CH	7.59 (t, 8.0, 7.3)
21	134.6, CH	7.88 (t, 8.0)	128.4, CH	7.53 (t, 8.0)	134.5, CH	7.87 (d, 8.2, 7.3)
22	127.1, CH	7.75 (d, 8.0)	133.9, CH	7.67 (t, 7.4)	127.1, CH	7.73 (d, 8.2)
23	147.8, C	-	128.4, CH	7.53 (t, 8.0)	147.9, C	-
24	-	-	130.3, CH	8.25 (overlap)	-	-
25	148.6, CH	8.57 (s)	-	-	149.2, CH	8.52 (s)
26	18.2, CH_3_	1.34 (s)	-	-	16.5, CH_3_	1.30 (s)
27	22.5, CH_3_	1.13 (s)	-	-	22.7, CH_3_	1.28 (s)
28	174.0, C	-	-	-	-	-
29	33.0, CH	2.16 (s)	-	-	-	-
30	18.1, CH_3_	0.76 (d, 6.4)	-	-	-	-
31	18.6, CH_3_	0.52 (d, 7.0)	-	-	-	-
8-OMe	-	-	51.7, CH_3_	3.24 (s)	-	-
9-OH	-	-	-	6.31 (s)	-	-

Pseurotin I (**2**): White amorphous powder; ${\left[\text{α}\right]}_{D}^{25}$ −5.6 (*c* 0.50, MeOH); UV (MeOH) λ_max_ (log *ε*) 204 (4.03), 252 (3.81), 280 (3.57) nm; CD (0.11 mM, MeOH) λ_max_ (Δε) 210 (+18.00), 232 (−4.70), 250 (+5.58), 278 (−32.22), 314 (+8.18) nm; IR (KBr) *ν*_max_ 3,370, 2,958, 2,929, 2,850, 1,723, 1,703, 1,627, 1,449, 1,263, 1,104 cm^−1^; ^1^H and ^13^C NMR data (see Table 1);HRESIMS *m/z* 468.1623 [M + Na]^+^ (calcd for C_23_H_27_NNaO_8_, 468.1629).

Tryptoquivaline Z (**3**): White amorphous powder; ${\left[\text{α}\right]}_{D}^{25}$ +135 (*c* 0.10, MeOH); UV (MeOH) λ_max_ (log *ε*) 208 (4.33), 296 (3.28) nm; CD (0.11 mM, MeOH) λ_max_ (Δε) 222 (−11.10), 244 (−2.27), 258 (−4.95), 302 (+1.30) nm; IR (KBr) *ν*_max_ 3,359, 2,932, 1712, 1,661, 1,611, 1,483, 1,465, 1,403, 1,293, 1,271, 1,172, 1,122, 1,019, 904, 758, 701 cm^−1^; ^1^H and ^13^C NMR data (see Table 1); HRESIMS *m/z* 451.1622 [M + H]^+^ (calcd for C_23_H_23_N_4_O_6_, 451.1612).

### Computational Details

All the quantum mechanical calculations were performed using Gaussian 09 (Frisch et al., 2009). Systematic conformational searches were done for each compound in the gas phase using the MMFF force field, implemented in Spartan 14 (Spartan’14), and the results were validated using Macromodel (MacroModel, 2018) (MMFF force field, mixed torsional/low-mode sampling protocol) using an energy cutoff of 10 kcal/mol. The choice for the 10 kcal/mol of cutoff was set as a balance between reducing the overall CPU calculation time and minimizing the possibility of losing further contributing conformers. The numbers of unique conformations found within these boundaries were 92 for **1**, 73 for *11epi*-**1**, and 290 for **2**. All conformers were kept for full geometry optimization at the RHF/3-21G level in gas phase. All structures within 5 kcal/mol from the corresponding global minima were reoptimized at the B3LYP/6-31G* level in gas phase. Frequency calculations were done at the same level to determine the nature of the stationary points found. The ECD calculations were carried out using the B3LYP/6-31G* optimized geometries. The excitation energies (nm) and rotatory strength (*R*) in dipole velocity (*R*_*vel*_) of the first forty singlet excitations were calculated using TDDFT implemented in Gaussian 09 at the PBE0/def2-SVP and B3LYP/6-31G* levels from all significantly populated conformers, which were averaged using Boltzmann weighting. The Boltzmann amplitudes obtained by refining the Gibbs free energies of all compounds at the SMD/M06-2X/6-31G* level using methanol as solvent. The calculated rotatory strength were simulated into the ECD curve as the sum of Gaussians with 0.3 eV width at half-heights (*σ*), which were UV-corrected and scaled (Pescitelli and Bruhn., 2016).

### Antibacterial Assays

Antibacterial assay was conducted by the previously described method (Cheng et al., 2013). DMSO (5%) was used as negative controls whereas chloramphenicol was used as a positive control, which was active against *S. aureus, methicillin resistant S. aureus, Bacillus subtilis* and *E. coli* with MIC values ranging from 2.5 μg/ml to 12.5 μg/ml. The maximum concentration of the used compounds was 160 μg/ml. All experiments were repeatedly performed in triplicate.

### Anti-Proliferative Assays

The viability of A2780 human ovarian cancer cells was determined using the CyQuant assay according to the manufacturer’s instructions (Life Technologies, CA, United States). Briefly, cells were cultured in 96-well plates at 5 × 10^3^ cells per well for 24 h and subsequently treated with compounds (50 μM) for 72 h and analyzed. Relative viability of the treated cells was normalized to the DMSO-treated control cells (Cao et al., 2007; Cao et al., 2010; Hou et al., 2008. Cisplatin was used as a positive control, which had an IC_50_ value of 0.36 μM. All experiments were performed in triplicate.

### NF-κB Assay

We employed human embryonic kidney cells 293, Panomic for monitoring changes occurring along the NF-κB pathway (Li et al., 2017). Stable constructed cells were seeded into 96-well plates at 20 × 10^3^ cells per well. Cells were maintained in Dulbecco’s modified Eagle’s medium (DMEM) (Invitrogen Co.), supplemented with 10% FBS, 100 units/mL penicillin, 100 μg/ml streptomycin, and 2 mM l-glutamine. After 48 h incubation, the medium was replaced and the cells were treated with various concentrations of test substances. TNF-α (human, recombinant, *E. coli*, Calbiochem) was used as an activator at a concentration of 2 ng/ml (0.14 nM). The plate was incubated for 6 h. Spent medium was discarded, and the cells were washed once with PBS. Cells were lysed using 50 μL (for 96-well plate) of reporter lysis buffer from Promega by incubating for 5 min on a shaker, and stored at −80°C. The luciferase assay was performed using the Luc assay system from Promega. The gene product, luciferase enzyme, reacts with luciferase substrate, emitting light, which was detected using a luminometer (LUMIstar Galaxy BMG). Data for NF-κB inhibition are expressed as IC_50_ values (i.e., concentration required to inhibit TNF-induced NF-κB activity by 50%). The known NF-κB inhibitor TPCK was used as a positive control.

## Results and Discussion

### Identification of Compounds

**FIGURE 1:**
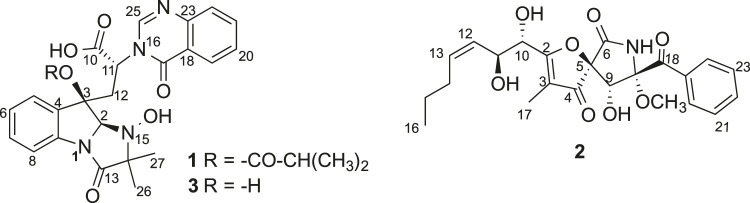
Chemical structures of compounds 1–3.

**FIGURE 2:**
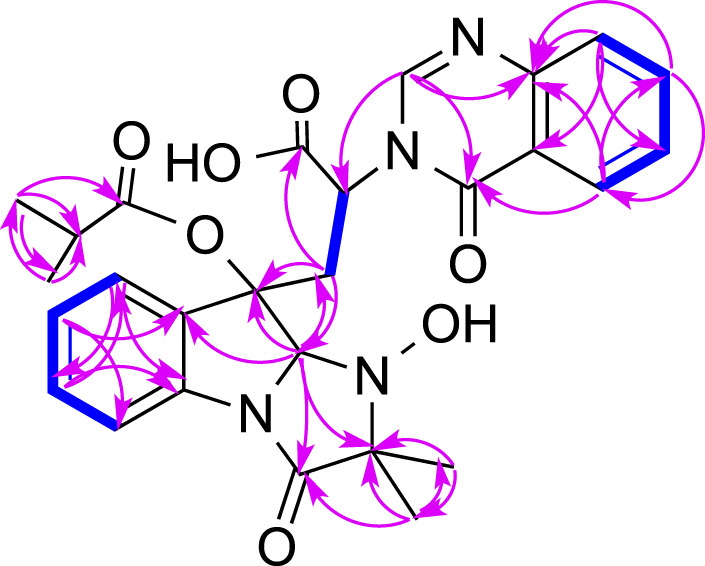
Key COSY (bolds, blue), HMBC (arrows, pink) and ROESY (double arrows, pink) correlations of **1**.

Compound **1** (Figure 1) was obtained as a white amorphous powder and its molecular formula was determined as C_27_H_28_N_4_O_7_ by HRESIMS, indicating sixteen degrees of unsaturation. The ^13^C NMR and HSQC spectra of **one** demonstrated the presence of nineteen carbons including four methyl (4× CH_3_), nine *sp* ^2^ methines (9× =CH), four *sp* ^2^ non-protonated carbons (4× = C), four carbonyls (4× -CO), and one methylenes (1×-CH_2_), three *sp* ^3^ methines (3× -CH), one nitrogenated nonprotonated *sp* ^3^ carbon (1× -C), and one oxygenated nonprotonated *sp* ^3^ carbon (1× -C) (Table 1). The COSY spectrum of **1** indicated the presence of three spin systems including one CH-CH_2_ and two CH = CH-CH = CH (Figure 2). The HMBC spectrum of **1** showed long-range ^1^H−^13^C correlations from H-5 (δ_H_ 7.36) to C-7 (δ_C_ 130.0) and C-9 (δ_C_ 133.6), from H-6 (δ_H_ 7.09) to C-4 (δ_C_ 139.8) and C-8 (δ_C_ 114.9), from H-7 (δ_H_ 7.35) to C-5 (δ_C_ 123.8) and C-9, from H-27 (δ_H_ 1.13) to C-14 (δ_C_ 69.4) and C-26 (δ_C_ 18.2), from H-26 (δ_H_ 1.34) to C-13 (δ_C_ 173.1) and C-14, and from H-2 (δ_H_ 5.08) to C-3 (δ_C_ 84.1), C-4, C-13, and C-14 (Figure 2), which confirmed the presence of an indole imidazole moiety, 2,2-dimethyl-1,2,9,9a-tetrahydro-3*H*-imidazo [1,2-*a*]indol-3-one. In the HMBC spectrum of **1**, H-22 (δ_H_ 7.75) correlated to C-20 (δ_C_ 127.1) and C-18 (δ_C_ 121.6), H-21 (δ_H_ 7.88) to C-19 (δ_C_ 126.1) and C-23 (δ_C_ 147.8), H-19 (δ_H_ 8.19) to C-21 (δ_C_ 134.6) and C-17 (δ_C_ 160.2), H-25 (δ_H_ 8.57) to C-17 and C-23, and H-25 to C-17 and C-22 (δ_C_ 127.1), which confirmed the presence of a quinazolin-4(3*H*)-one moiety. The HMBC correlations from H-12 (δ_H_ 2.81, 3.15) to C-2 (δ_C_ 85.1), C-3 and C-10 (δ_C_ 170.1), and H-25 and C-11 indicated that the indole imidazole and quinazolin-4(3*H*)-one moieties were connected through the ^12^CH_2_-^11^CH spin system with a carboxylic acid group at 11-position.The HMBC spectrum of **1** also showed long-range ^1^H−^13^C correlations from H-31 (δ_H_ 0.52) to C-29 (δ_C_ 33.0) and C-30 (δ_C_ 18.1), H-30 (δ_H_ 0.76) to C-28 (δ_C_ 174.0), C-29 and C-31 (δ_C_ 18.6), indicating the presence of an isobutyric acid group in compound **1**. On the basis of the NOESY correlations from H-30 to H-25 (δ_H_ 8.57) and H-31 to H-5, the isobutyric acid group must be located at 3-position (Figure 2). Finally, the hydroxyl group was assigned at 15-position because this was the only available position. Hence, the planar structure of **one** was determined as shown, and it was named tryptoquivaline Y. The ROESY spectrum of compound **1** exhibited correlations between H-2 (δ_H_ 5.08) and H-12 (δ_H_ 2.81), indicating that H-2 and H-12 were on the same side of the molecule.

**FIGURE 3:**
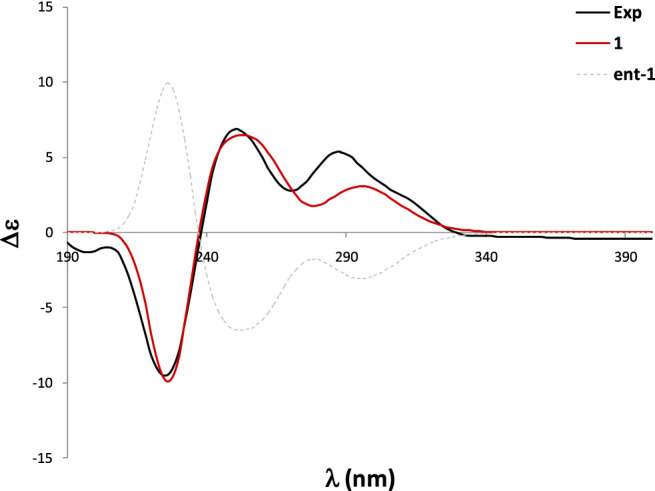
Experimental and calculated ECD spectra of **1**.

In order to determine the absolute configuration of **1**, a CD spectrum was collected, which was very similar to that of tryptovaline K (Zhou et al., 2012), indicating that both compounds should have the same absolute configuration. To confirm the absolute configuration of **1**, TDDFT ECD calculations were carried out. The experimental ECD of **1** showed a strong negative Cotton effect (CE) at 224 nm, and two positive CEs at 250 and 288 nm. The MMFF conformational analysis of **1** yielded 92 conformations within the 10 kcal/mol window, which were further subsequently reoptimized at the RHF/3-21G and B3LYP/6-31G* levels. The Gibbs free energies of the most stable conformations found were further refined at the SMD/M06-2X/6-31G* level of theory, using methanol as solvent. The ECD calculations were performed at the PBE0/def2SVP//B3LYP/6-31G* level, and were Boltzmann-averaged using the Gibbs free energies calculated in the previous step. The same computational work was carried out with *11epi* **-1** in order to define the relative configuration at C-11 as well. As shown in Figure 3, the calculated ECD of **1** showed an excellent agreement with the experimental data, allowing to assign the structure of **1** as shown. On the other hand, the calculated spectrum of *11epi* **-1** did not reflect good match with the experimental data (Supplementary Figures S22, 23), hence reinforcing our relative and absolute configurational assignment.

**FIGURE 4:**
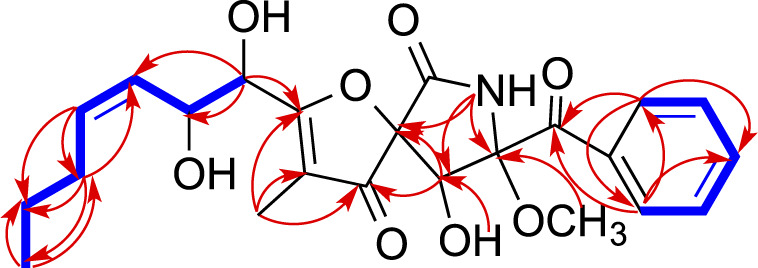
Key COSY (bolds, blue), HMBC (arrows, red) correlations of **2**.

**FIGURE 5:**
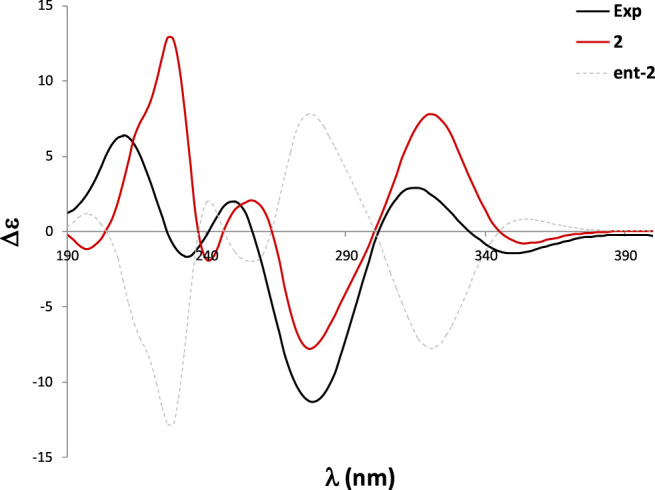
Experimental and calculated ECD spectra of **2**.

Pseurotin F (**2**) was obtained as white amorphous powder and has a molecular formula of C_23_H_27_NO_8_ derived from the HRESIMS peak at *m/z* 468.1623 [M + Na]^+^. The COSY spectrum of **2** exhibited the presence of two spin system, CH_3_-CH_2_-CH_2_-CH = CH-CH(OH)-CH(OH)- and a mono-substituted benzene ring (Figure 4). HMBC correlations from H-17 (δ_H_ 1.63) to C2 (δ_C_ 187.0), and C-4 (δ_C_ 196.8), H-9 (δ_H_ 4.40) to C-4 and C-5 (δ_C_ 92.5), 9-OH (δ_H_ 6.31) to C-9 (δ_C_ 75.0), 8-OMe (δ_H_ 3.24) to C-8 (δ_C_ 91.2), and NH-7 (δ_H_ 9.95) to C-5, C-8, and C-9 indicated the presence of 1-oxa-7-azaspiro [4.4]non-2-ene-4,6-dione core. An HMBC correlation from H-10 (δ_H_ 4.33) to C-2 confirmed the connectivity of the spin system CH_3_-CH_2_-CH_2_-CH = CH-CH(OH)-CH(OH)- to the 1-oxa-7-azaspiro [4.4]non-2-ene-4,6-dione core. The mono-substituted benzene ring must be connected to the 1-oxa-7-azaspiro [4.4]non-2-ene-4,6-dione core through a ketone (C=O). Moreover, the NMR data of compound **2** (Table 1) were very similar to those of compound **8**. The main NMR difference between **2** and **8** was attributable to one more methylene group in the chain of compound **2**. No ROESY correlation between H-9 (δ_H_ 4.40, s) and 8-OMe (δ_H_ 3.24, s) was observed. In fact, no other ROESY correlations were clear except for those in the two spin systems. On the other hand, the ^13^C NMR data of compound **2** and compound **8** were almost the same, indicating both **2** and compound **8** should have the same configuration. Interestingly, compounds **2** and **8** had completely the same ECD pattern (Figure 5 and Supplementary Figure S14). Hence, it was confirmed that both compounds **2** and **8** should have the same configuration, and compound **2** was named pseurotin I.

To confirm the absolute configuration of **2**, we performed the ECD calculations. Starting with the 290 conformations found at the MMFF level, the geometries were subsequently reoptimized at the RHF/3-21G and B3LYP/6-31G* levels. The TDDFT calculations were carried at the B3LYP/6-31G* level (which in this case yielded better performance than the PBE0/def2SVP level) and were Boltzmann-averaged using the Gibbs free energies refined at the SMD/M06-2X/6-31G* level with methanol as solvent. The calculated ECD spectrum of **two** was in good agreement with the experimental ECD curve, which thus allowed an unambiguous configurational assignment (Figure 5).

**FIGURE 6:**
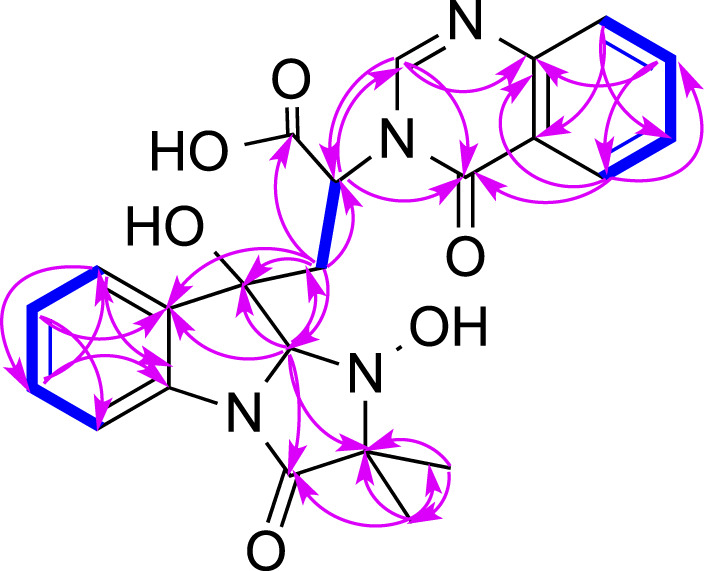
Key COSY (bolds, blue), HMBC (arrows, pink) correlations of **3**.

Tryptoquivaline Z (**3**) was obtained as white amorphous powder and has a molecular formula of C_23_H_22_N_4_O_6_ derived from the HRESIMS peak at *m/z* 451.1622 [M + H]^+^. The NMR data of compound **3** (Table 1) were very similar to those of compound **1**. The main NMR difference between **3** and **1** was that compound **1** had one extra isobutyric acid group (Figures 1, 6). Compound **3** had a similar CD spectrum to that of **1**, so it was deducted that both should have the same configuration. The planar structure of compound **3** was recorded in SciFinder with an ACS registration number of 1214809–50-3 (Zheng et al., 2018), but no physio-chemical properties including NMR data were reported in the published patent (Zheng et al., 2018). The ROESY spectrum of compound **3** showed correlations between H-2 (δ_H_ 4.88, s) and H-26 (δ_H_ 1.30, s), H-12 (δ_H_ 2.74, d) (Supplementary Figures S21), which were very similar to those of compound **1**. Further, compounds **3** and **1** had the similar ECD patterns, indicating that both compounds **3** and **1** should have the same configuration. Hence, the structure including the absolute configuration of compound **3** was determined as shown, and it was given a trivial name tryptoquivaline Z.

Seven other compounds, *β*-cyclopiazonic acid (**4**) (Wang et al., 2016), *cyclo*-(L-Pro-L-Phe) (**5**) (Li et al., 2008), tryptoquivaline L (**6**) (Buttachon et al., 2012), Bisdethiobis (methylthio) gliotoxin (**7**) (Afiyatullov et al., 2005), pseurotin A (**8**), pseurotin A_1_ (**9**) and pseurotin A_2_ (**10**) (Wang et al., 2011) were also isolated from *Aspergillus felis* FM324. The structures of these known compounds (**4**–**10**) were determined based on comparisons of NMR and HRESIMS data with previously reported data.

### Biological Activity

Except for compound **4**, the other nine compounds belong to three different classes of natural products, tryptoquivalines (**1**, **3**, and **6**), pseurotins (**2** and **8**–**10**) and diketopepirazines (**5** and **7**). These classes of compounds were reported to demonstrate anti-proliferative and antibacterial activities. Hence, we tested compounds **1**–**10** for their activities against A2780 cancer cell line, *S. aureus,* methicillin resistant *S. aureus, Bacillus subtilis* and *E. coli*. Besides, their anti-inflammatory activity against NF-κB was also evaluated. Compound **4** showed antibacterial activity against *S. aureus*, methicillin resistant *S. aureus* and *Bacillus subtilis* with the same MIC value of 59.2 µM. None of the compounds (**1**–**10**) exhibited any anti-proliferative activity against A2780, while compounds **3** and **2** inhibited NF-κB with IC_50_ values of 26.7 and 30.9 μM, respectively. In the absence of a cytotoxic response, inhibition of NF-κB activity suggests the potential of cancer chemoprevention.

## Conclusion

*Aspergillus* species are well known for producing tryptoquivaline and pseurotin types of compounds. Our research group previously reported two new tryptoquivaline from *Aspergillus terreus* (Zaman K. A. U. et al., 2020). Pseurotins, with 1-oxa-7-azaspiro [4.4]non-2-ene-4,6-dione core, were also isolated from another *Aspergillus species* (Wang et al., 2011). In our current research, one new tryptoquivaline (**1**) and one new pseurotin (**2**) together with eight known compounds (**3**–**10**) were isolated from a Hawaiian fungal strain *Aspergillus felis* FM324. The absolute configuration and physicochemical properties of compound **3** were also described for the first time. Compound **4** showed weak antibacterial activity against Gram-positive bacteria, and compounds **2** and **3** mildly inhibited NF-κB.

## Data Availability

The datasets presented in this study can be found in online repositories. The names of the repository/repositories and accession number(s) can be found below: GenBank under the accession no. MZ227547.
