# Supplementary material for: New Alkaloids From a Hawaiian Fungal Strain Aspergillus felis FM324
Source: Front Chem. 2021 Aug 9;9:724617. doi: 10.3389/fchem.2021.724617 (PMC8380829; doi:10.3389/fchem.2021.724617)
Supplement: Supplementary file 1 [file DataSheet1.PDF]

frontiers

Supplementary Material

Alkaloids from a Hawaiian fungal strain *Aspergillus felis* FM324

Cong Wang[1,2], Ariel M. Sarotti[3], KH Ahammad Uz Zaman[1], Xiaohua Wu[1], and Shugeng Cao [1*]

[1] Department of Pharmaceutical Sciences, Daniel K. Inouye College of Pharmacy, University of Hawai'i at Hilo, Hilo, Hawaii 96720, United States

[2] Key Laboratory of Chemistry and Engineering of Forest Products, State Ethnic Affairs Commission, Guangxi Key Laboratory of Chemistry and Engineering of Forest Products, Guangxi Collaborative Innovation Center for Chemistry and Engineering of Forest Products, School of Chemistry and Chemical Engineering, Guangxi University for Nationalities, Nanning 530006, China

[3]Instituto de Química Rosario (CONICET), Facultad de Ciencias Bioquímicas y Farmacéuticas, Universidad Nacional de Rosario, Suipacha 531, Rosario 2000, Argentina; sarotti@iquir-conicet.gov.ar

***Correspondence:**
Shugeng Cao
scao@hawaii.edu

List of Supporting Information

Content Page

Figure S1. HRESIMS spectrum of compound **1** 3
Figure S2. The ^{1}H-NMR spectrum of compound **1** in DMSO-d_6 4
Figure S3. The ^{13}C-NMR spectrum of compound **1** in DMSO-d_6 5
Figure S4. The HSQC spectrum of compound **1** in DMSO-d_6 6
Figure S5. The COSY spectrum of compound **1** in DMSO-d_6 7
Figure S6. The HMBC spectrum of compound **1** in DMSO-d_6 8
Figure S7. The ROESY spectrum of compound **1** in DMSO-d_6 9
Figure S8. HRESIMS spectrum of compound **2** 10
Figure S9. The ^{1}H-NMR spectrum of compound **2** in DMSO-d_6 11
Figure S10. The ^{13}C-NMR spectrum of compound **2** in DMSO-d_6 12
Figure S11. The HSQC spectrum of compound **2** in DMSO-d_6 13
Figure S12. The COSY spectrum of compound **2** in DMSO-d_6 14
Figure S13. The HMBC spectrum of compound **2** in DMSO-d_6 15
Figure S14. The ROESY spectrum of compound **2** in DMSO-d_6 16
Figure S15. Experimental ECD spectra of **2** and **8** 17
Figure S16. HRESIMS spectrum of compound **3** 18
Figure S17. The ^{1}H-NMR spectrum of compound **3** in DMSO-d_6 19
Figure S18. The ^{13}C-NMR spectrum of compound **3** in DMSO-d_6 20
Figure S19. The HSQC spectrum of compound **3** in DMSO-d_6 21
Figure S20. The COSY spectrum of compound **3** in DMSO-d_6 22
Figure S21. The HMBC spectrum of compound **3** in DMSO-d_6 23
Figure S22. The ROESY spectrum of compound **3** in DMSO-d_6 24
Figure S23. B3LYP/6-31G* optimized geometries of the most stable conformers found for **1**, *11epi*-**1** and **2** 25
Figure S24. Experimental ECD of **1** and calculated ECD of *11epi*-**1** 26
Figure S25. Structures of compounds **4-10** 26

Cartesian coordinates of all compounds under study

Figure S1. HRESIMS spectrum of compound **1**

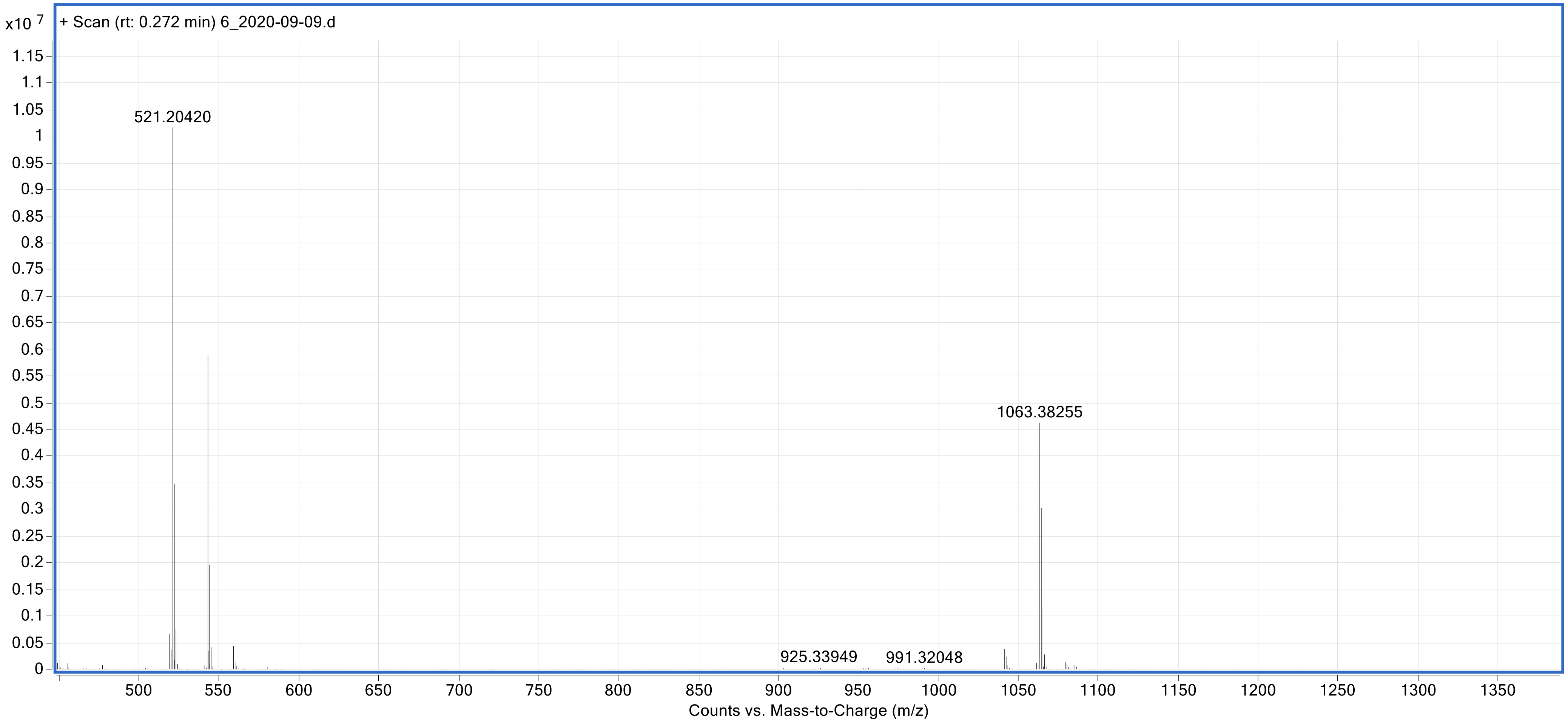

Figure S2. The ^{1}H-NMR spectrum of compound **1** in DMSO-d_6

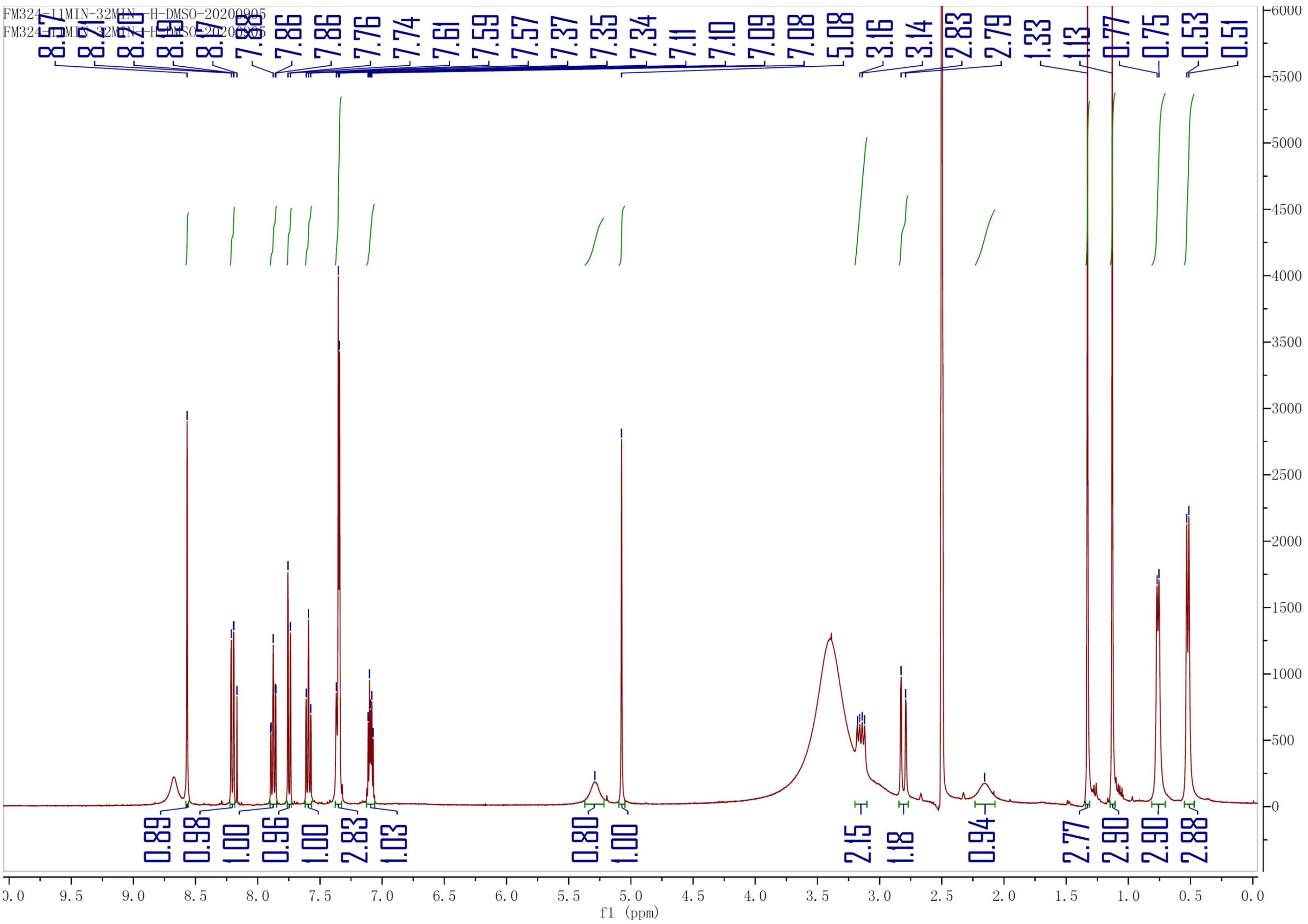

Figure S3. ^{13}C-NMR spectrum of compound **1** in DMSO-d_6

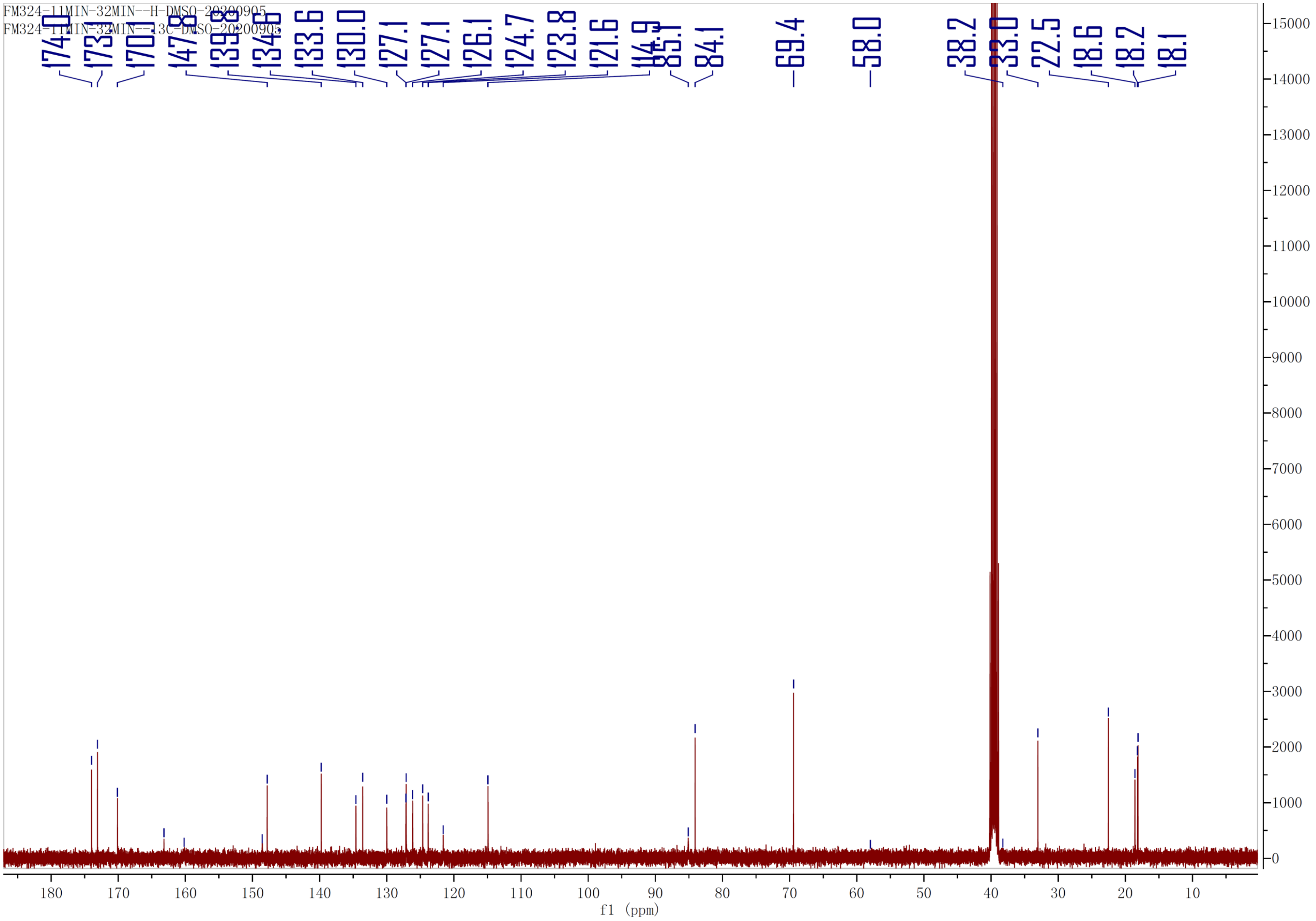

Figure S4. HSQC spectrum of compound **1** in DMSO-d_6

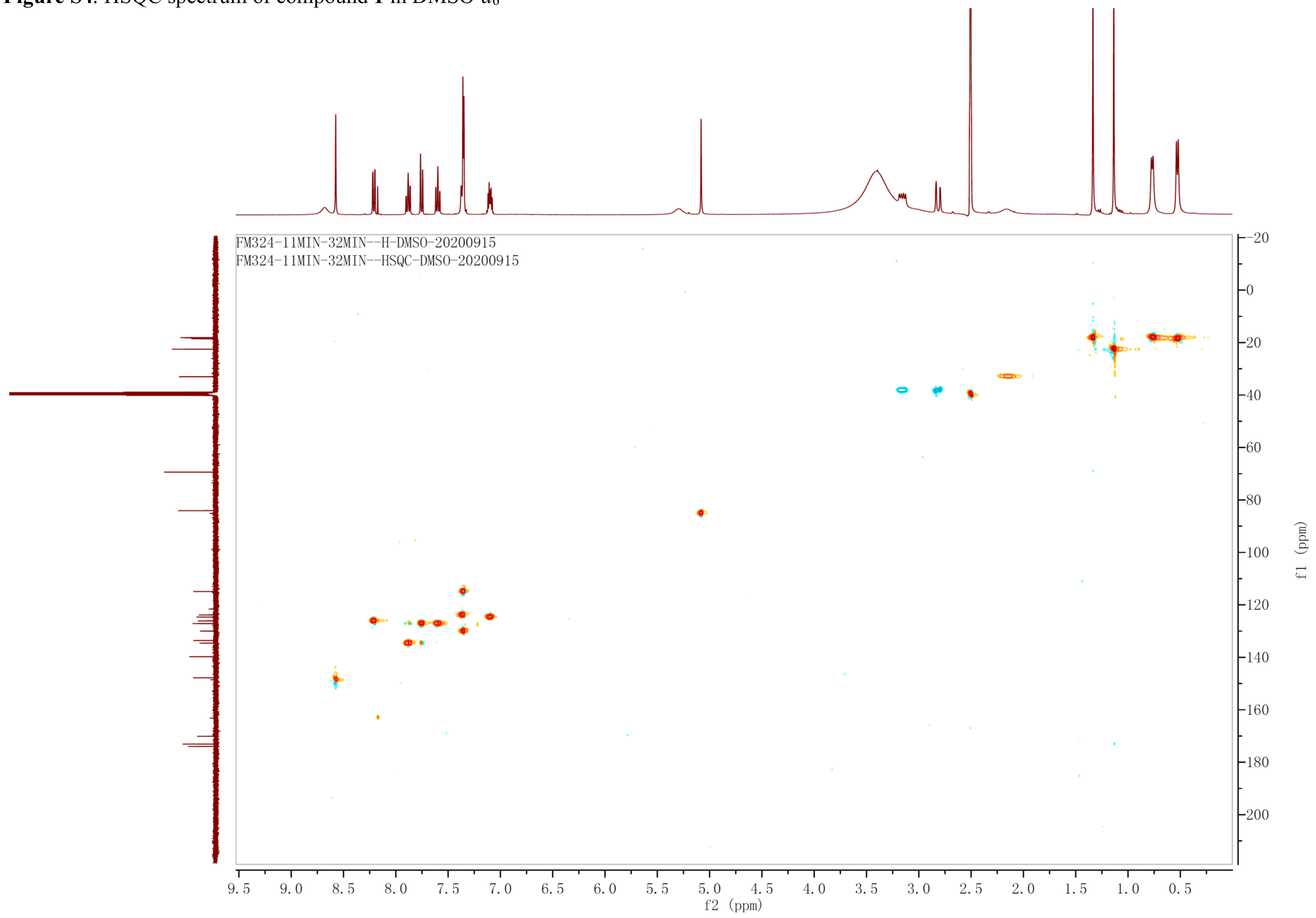

Figure S5. COSY spectrum of compound **1** in DMSO-d_6

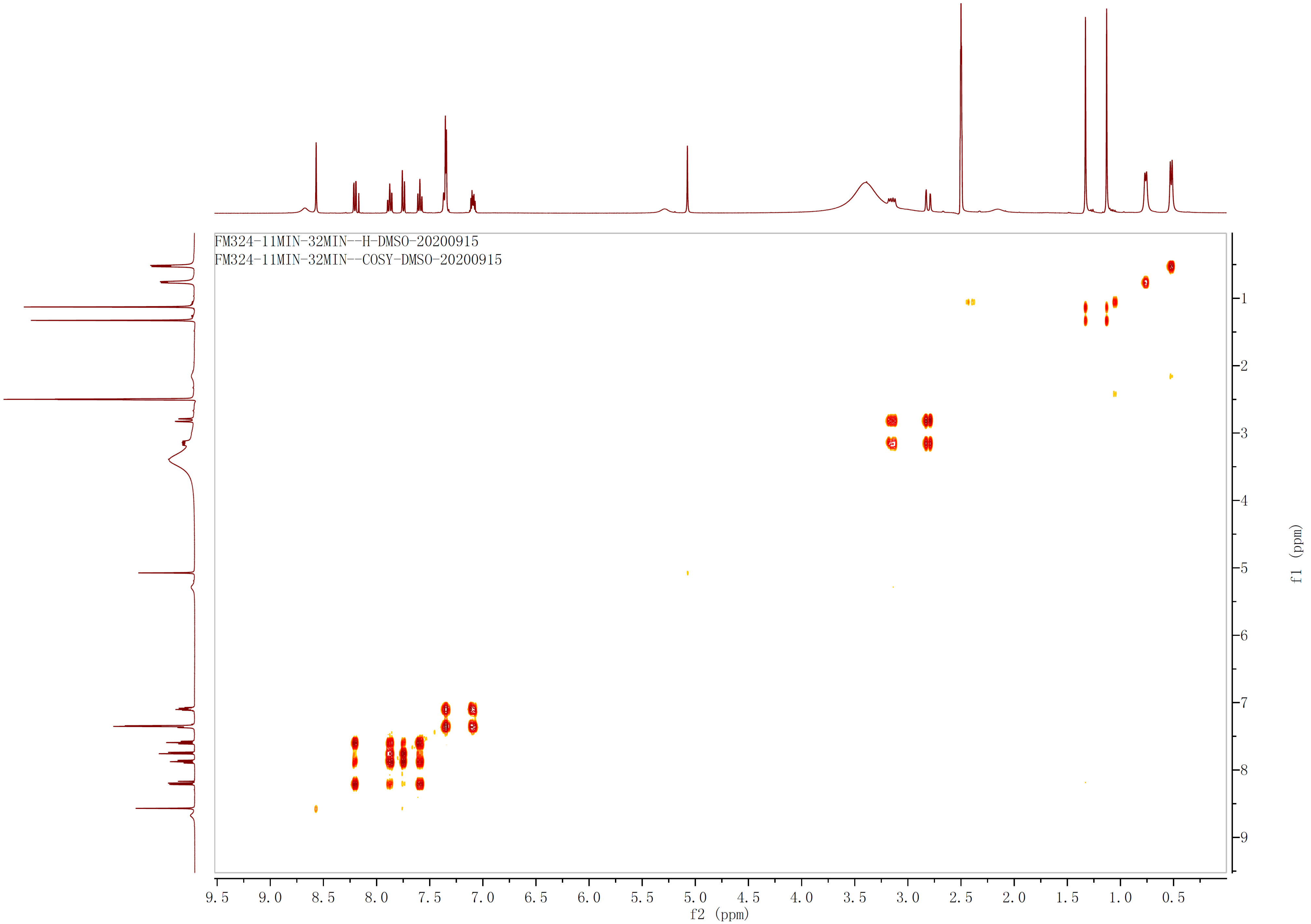

Figure S6. HMBC spectrum of compound **1** in DMSO-d_6

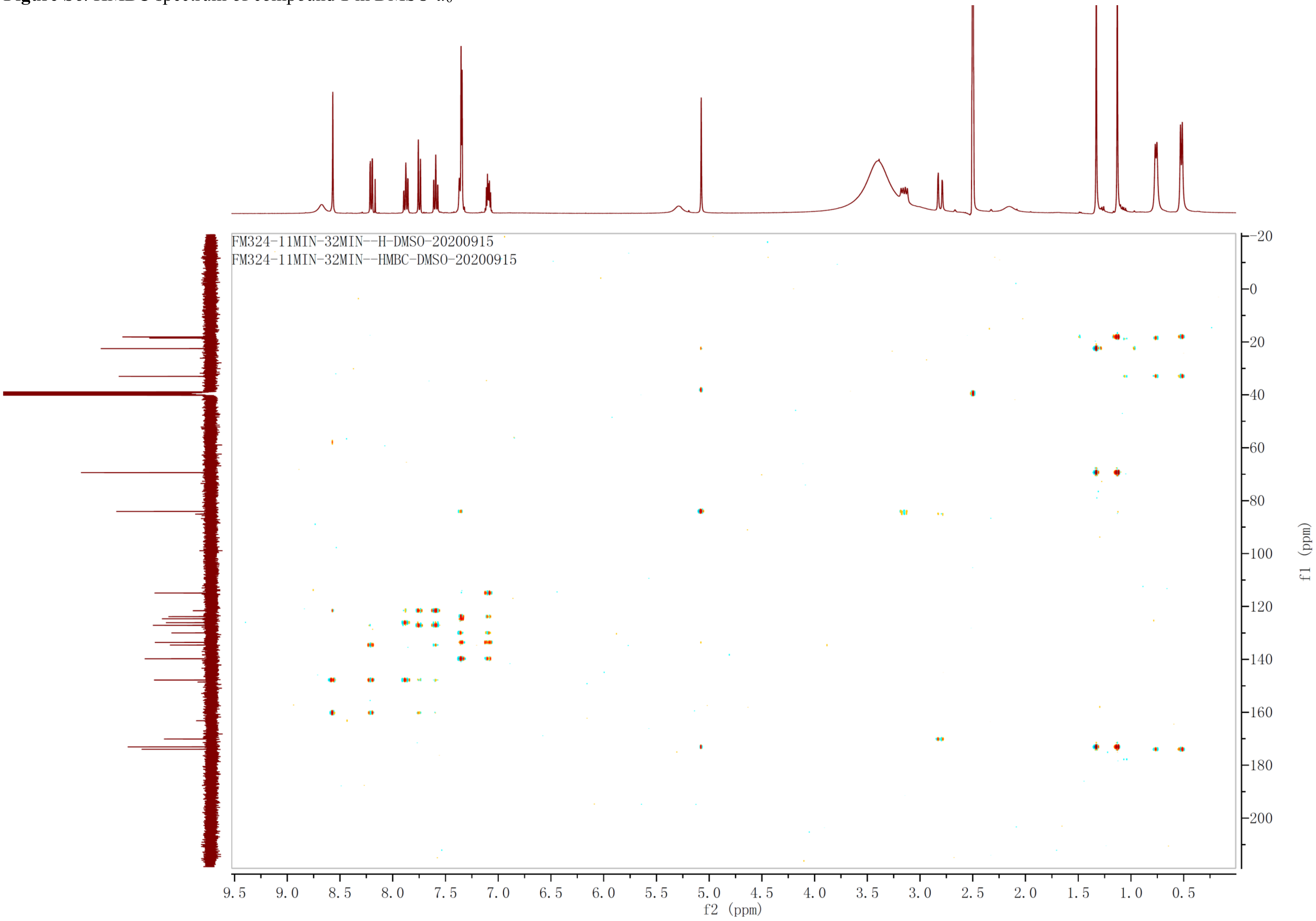

Figure S7. ROESY spectrum of compound **1** in DMSO-d_6

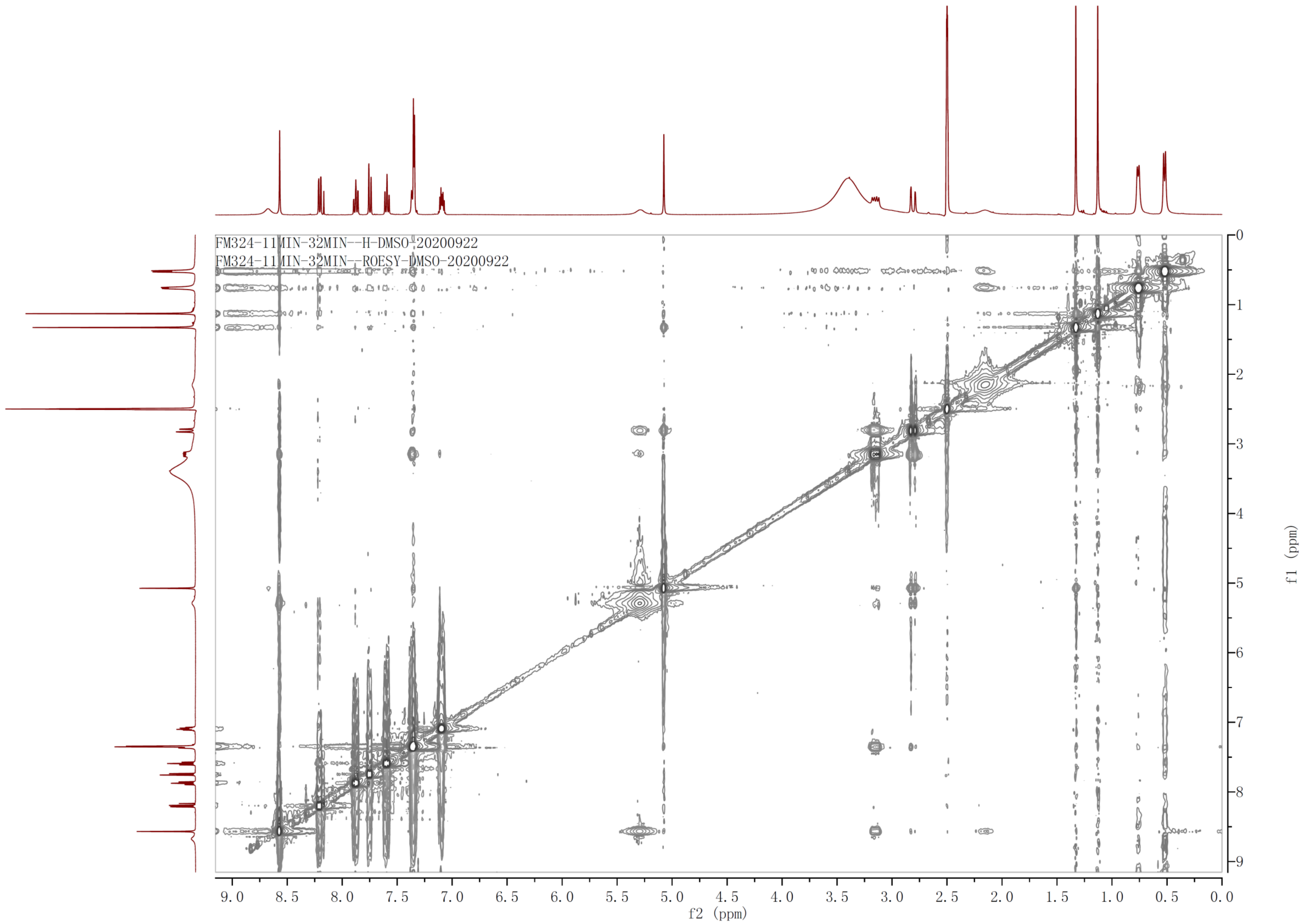

Figure S8. HRESIMS spectrum of compound **2**

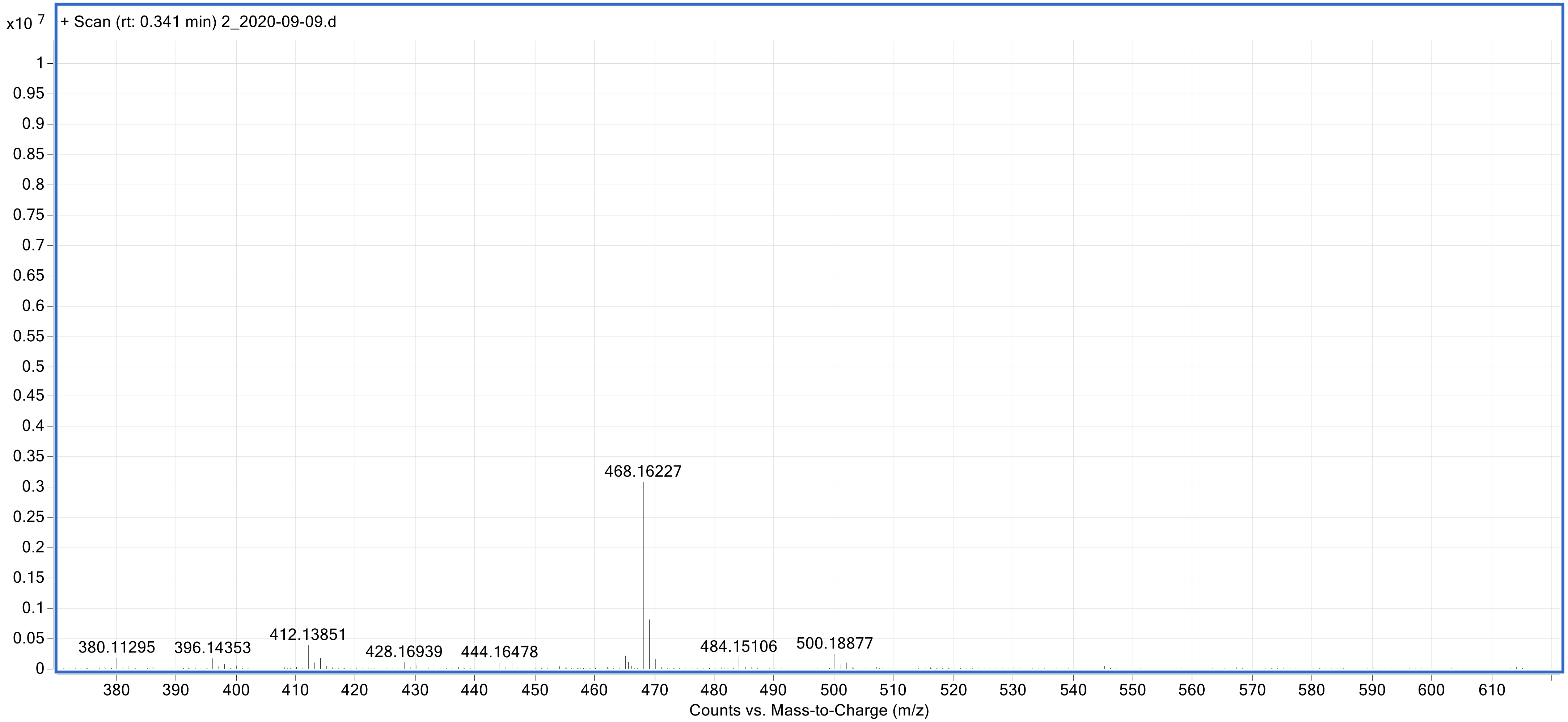

Figure S9. ^{1}H-NMR spectrum of compound **2** in DMSO-d_6

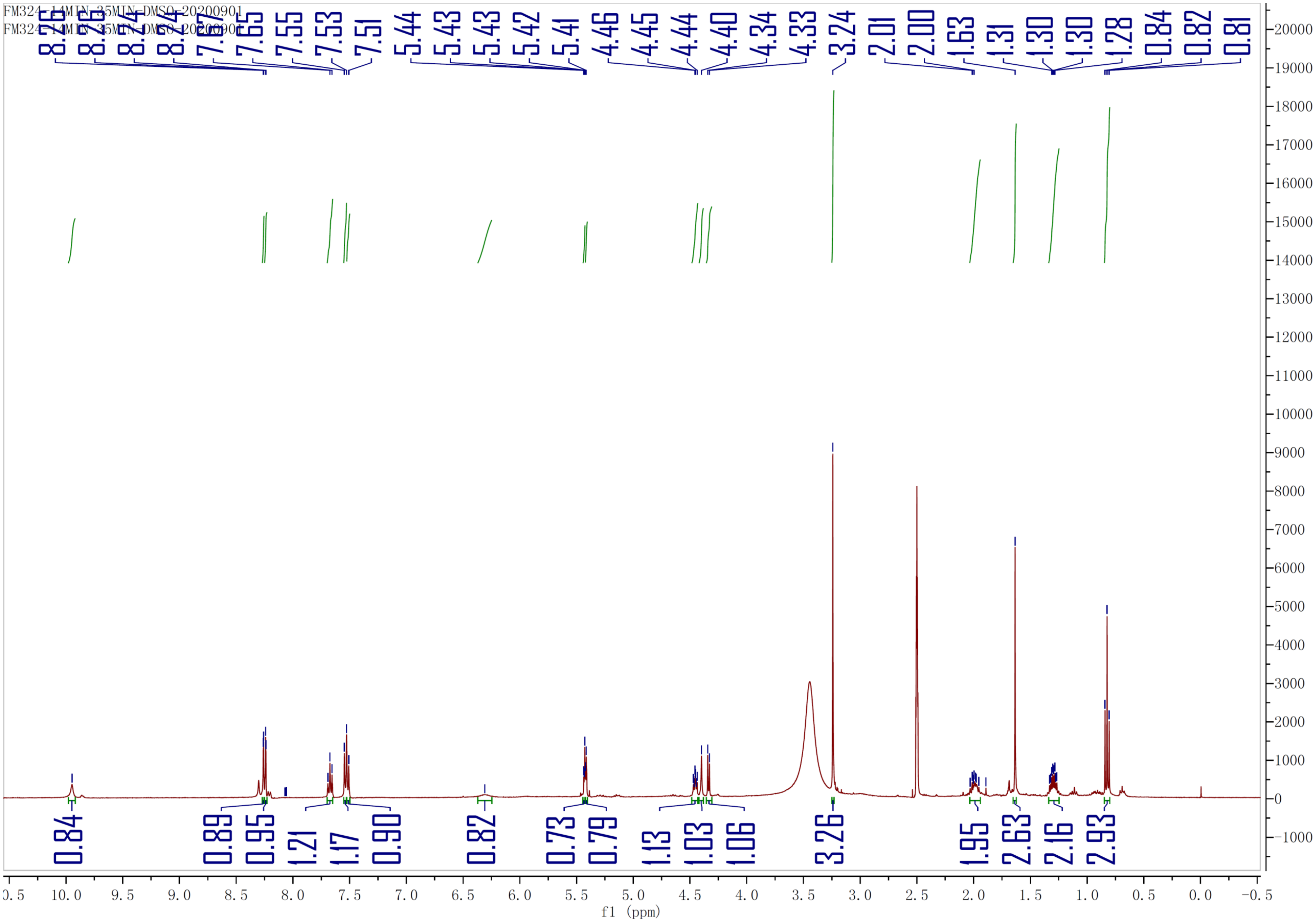

Figure S10. ^{13}C-NMR spectrum of compound **2** in DMSO-d_6

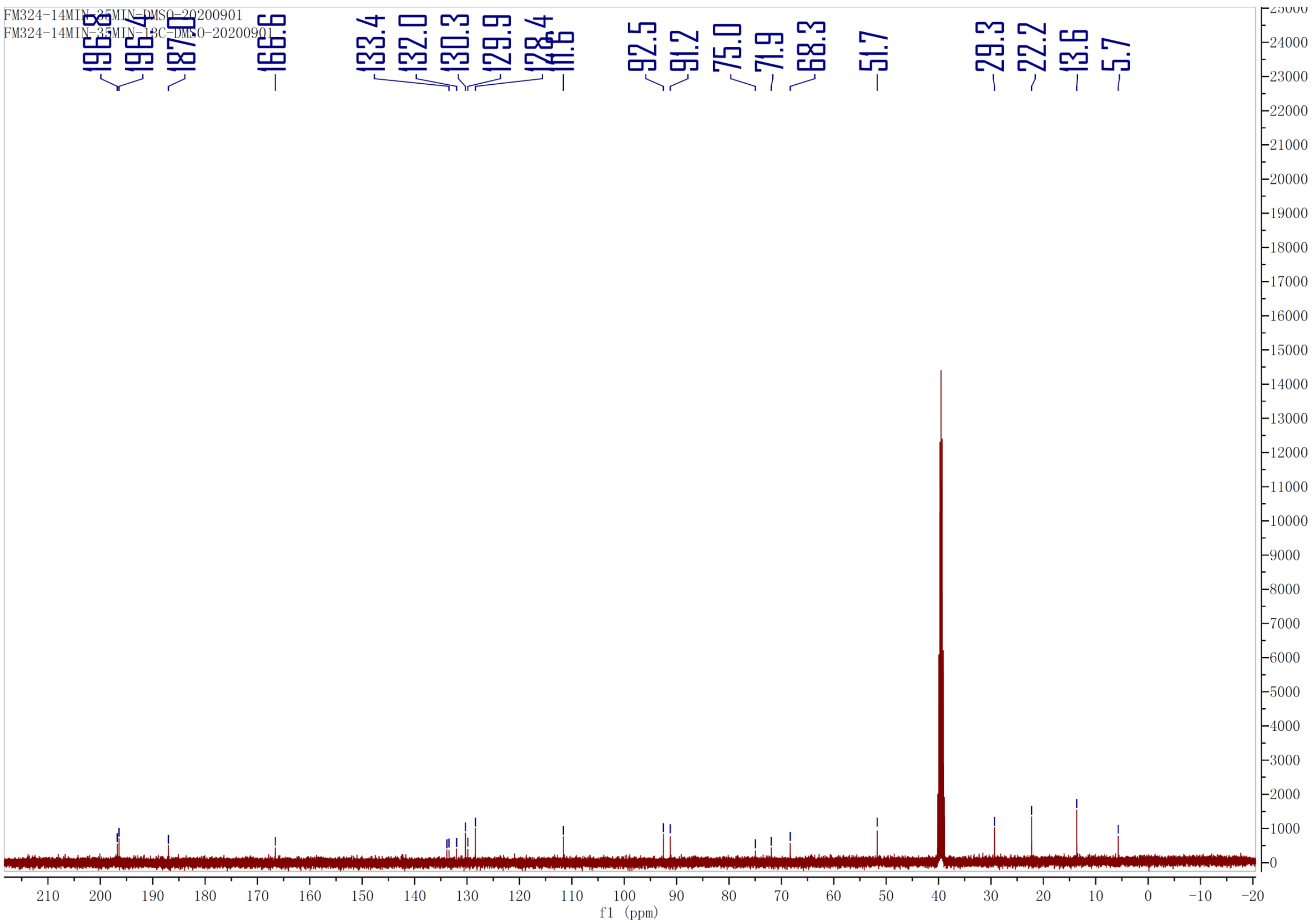

Figure S11. HSQC spectrum of compound **2** in DMSO-d_6

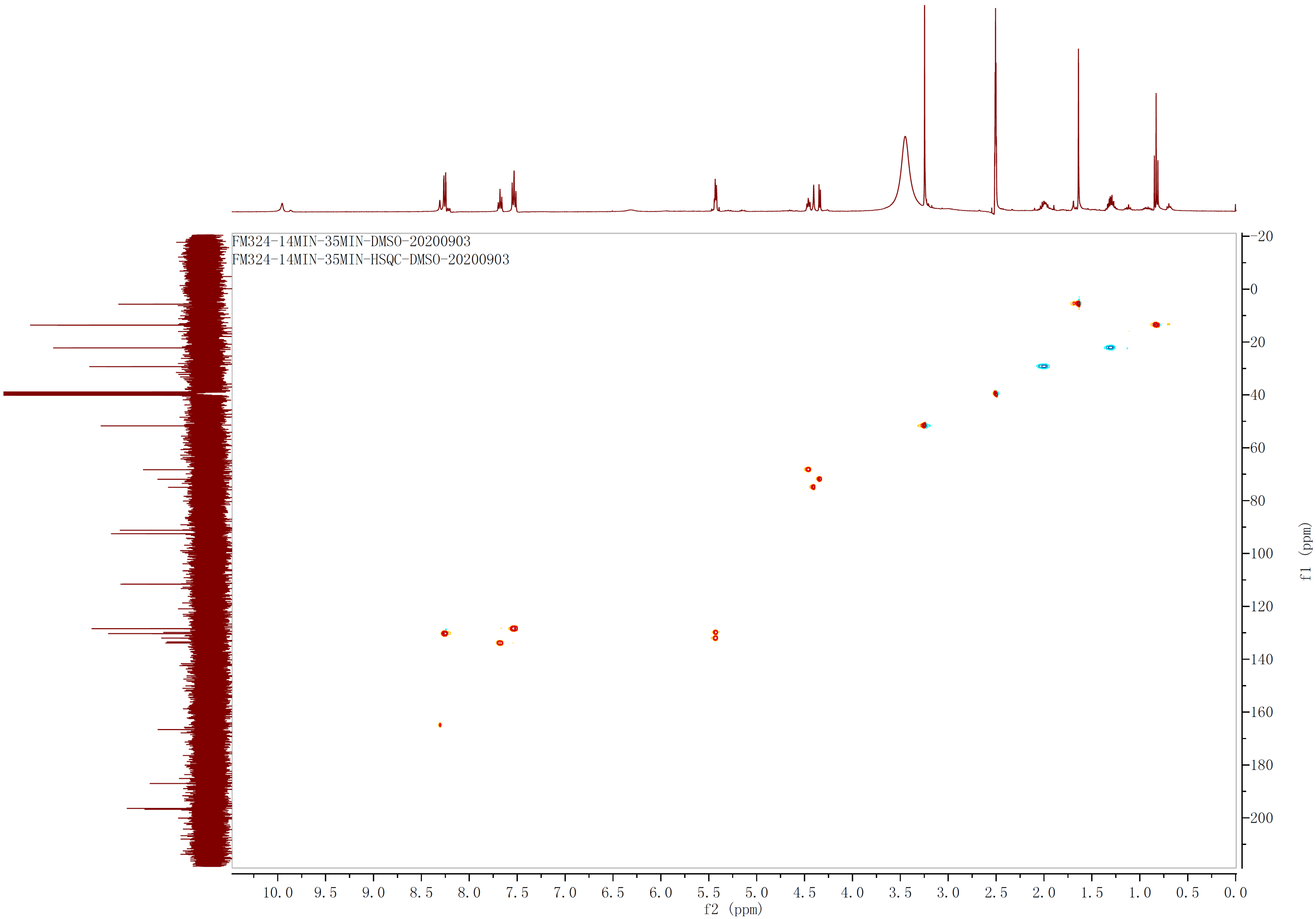

Figure S12. COSY spectrum of compound **2** in DMSO-d_6

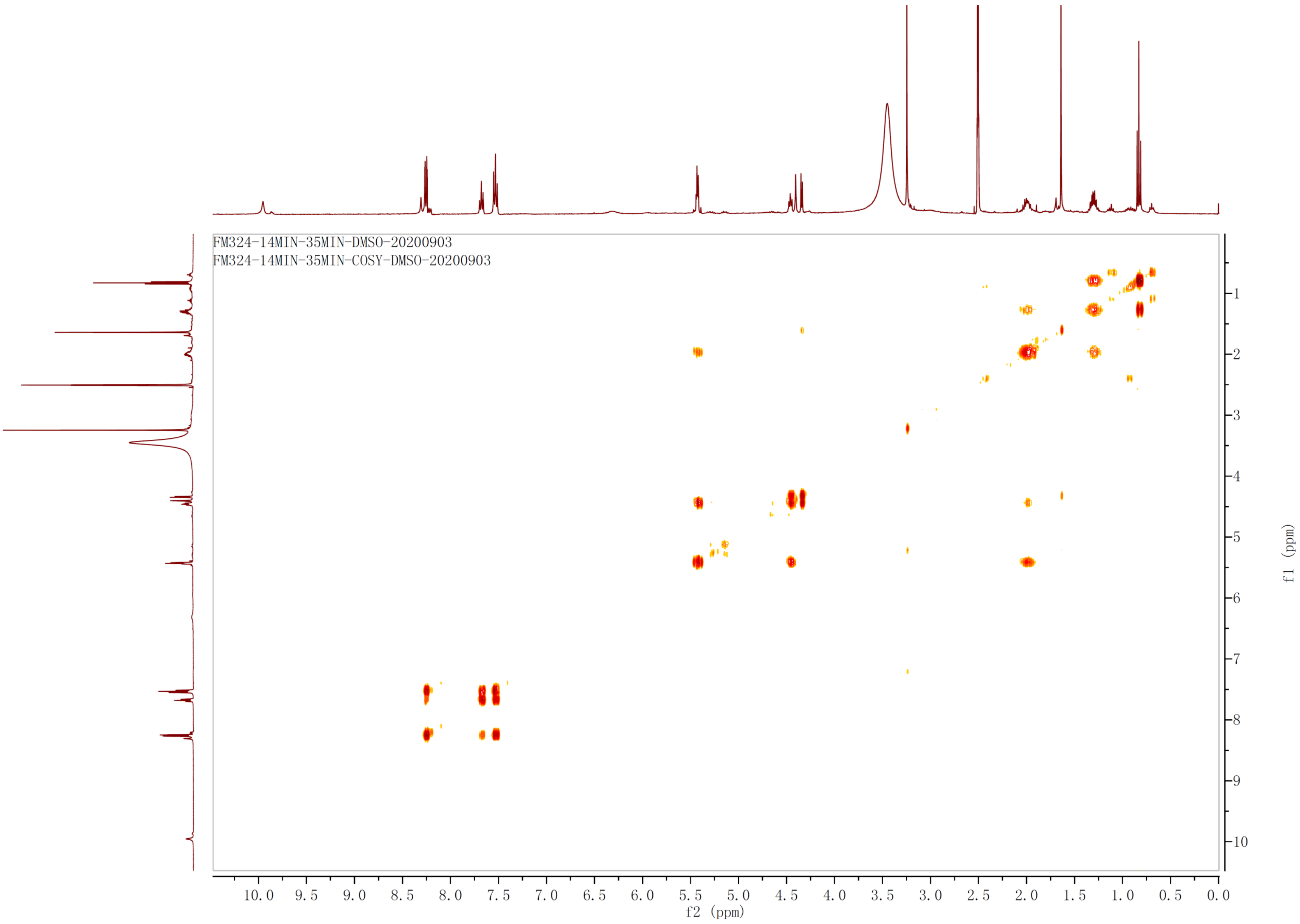

Figure S13. HMBC spectrum of compound **2** in DMSO-d_6

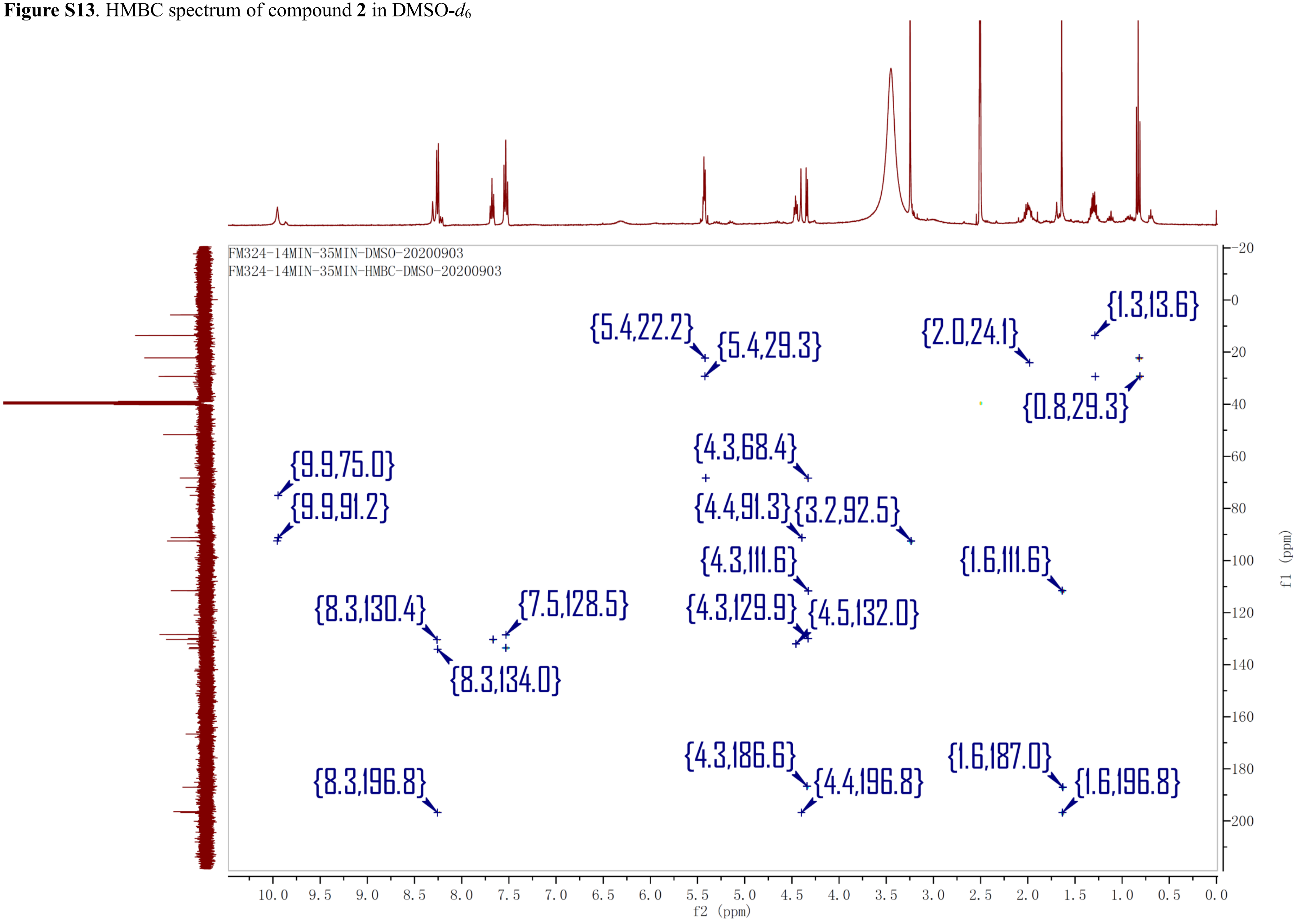

Figure S14. The ROESY spectrum of compound **2** in DMSO-d_6

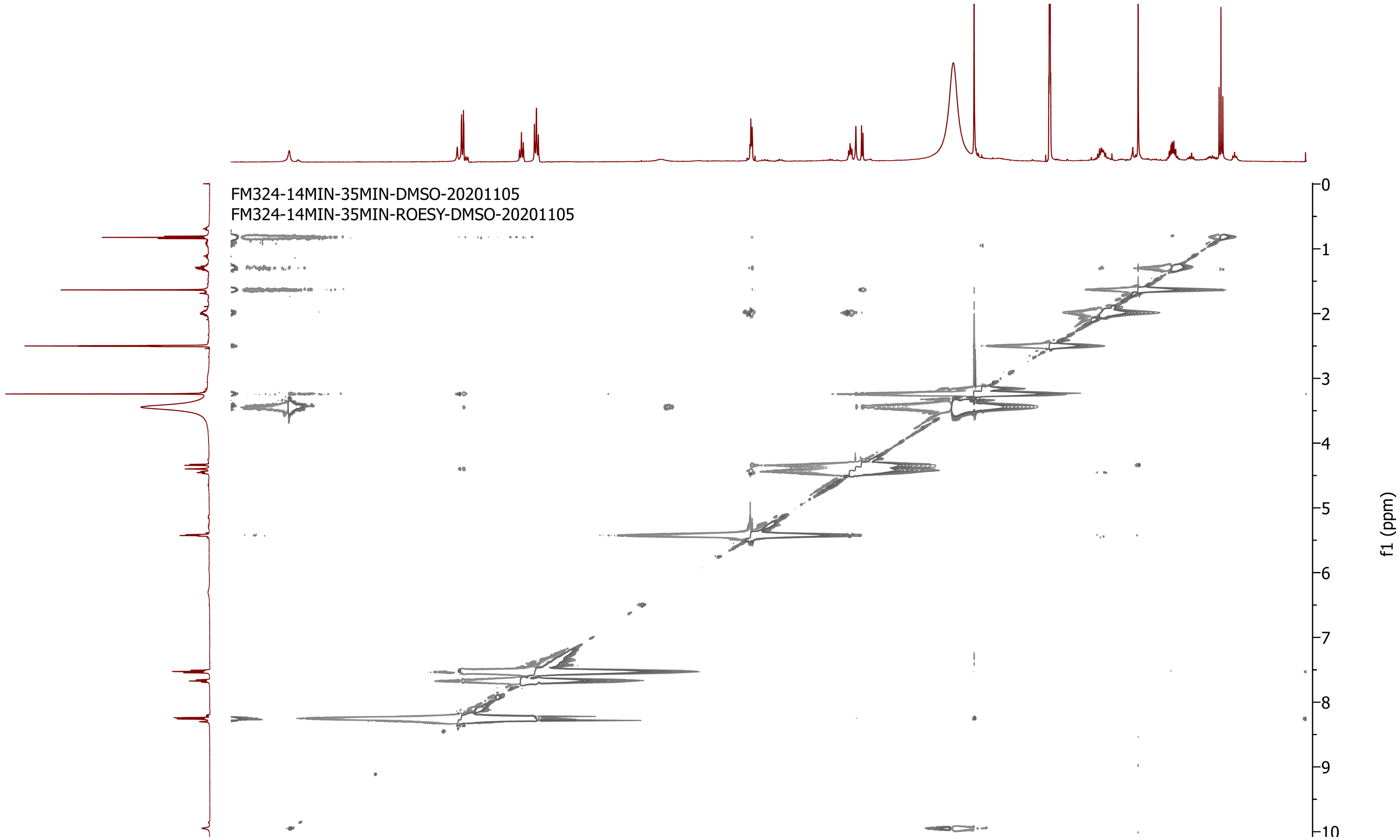

Figure S15. Experimental ECD spectra of **2** and **8**

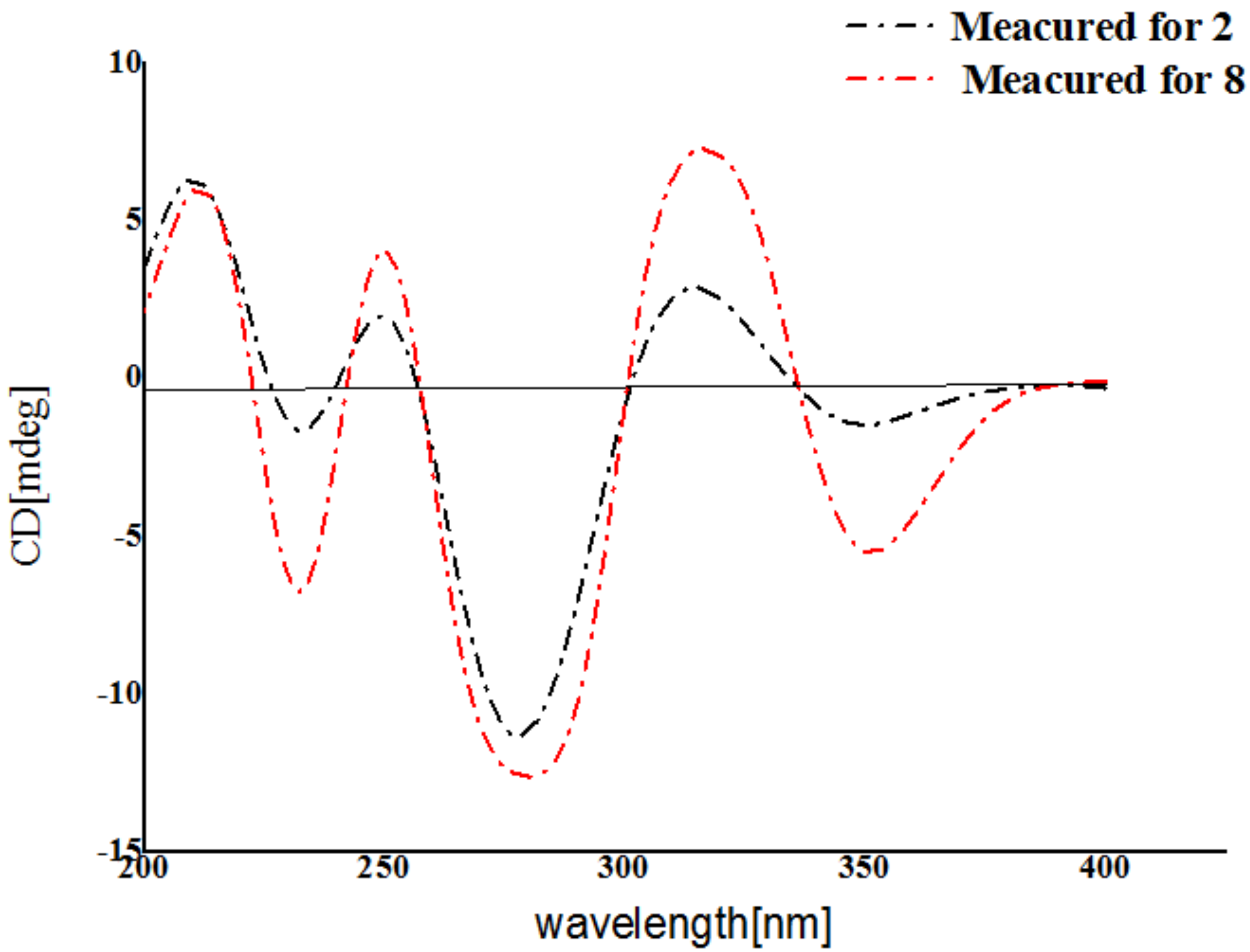

Figure S16. HRESIMS spectrum of compound **3**

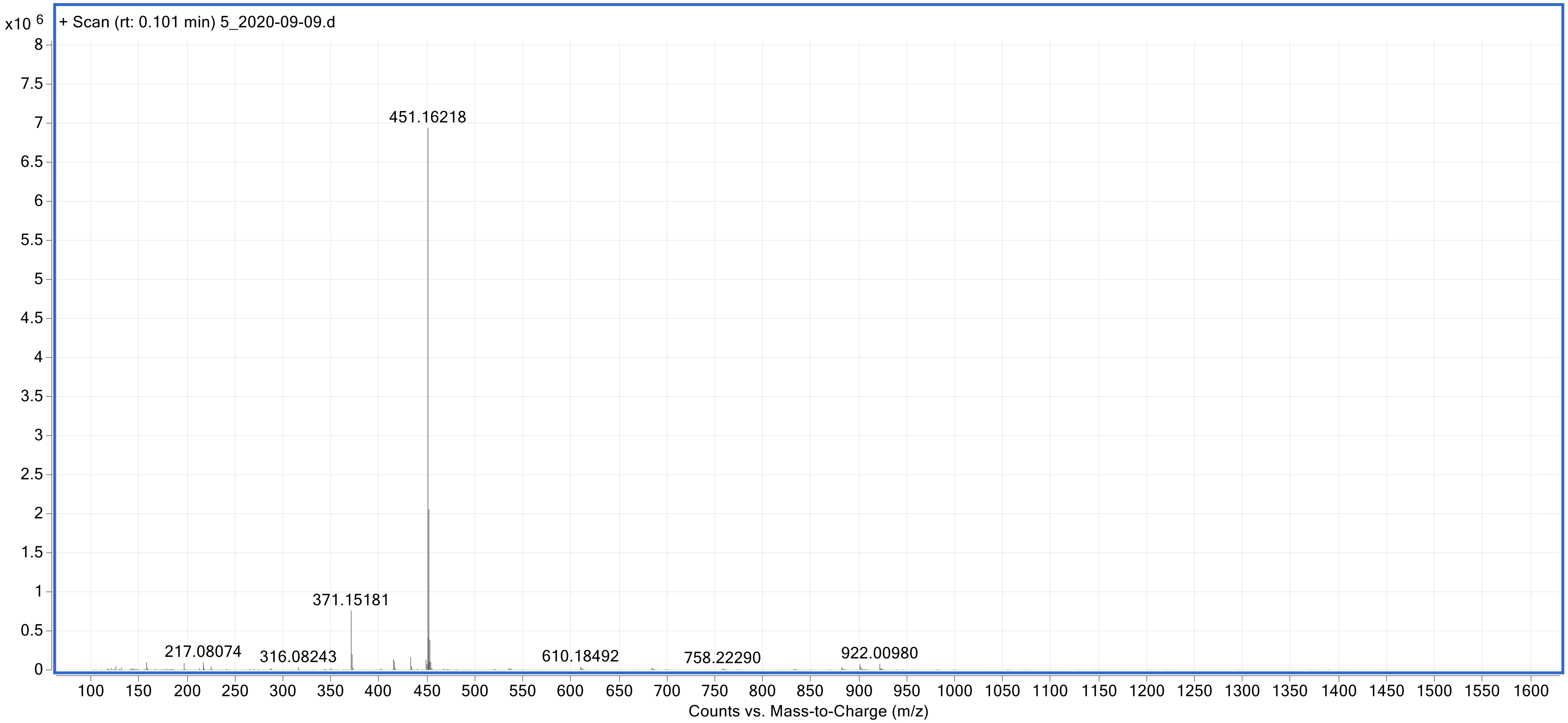

Figure S17. ^{1}H-NMR spectrum of compound **3** in DMSO-d_6

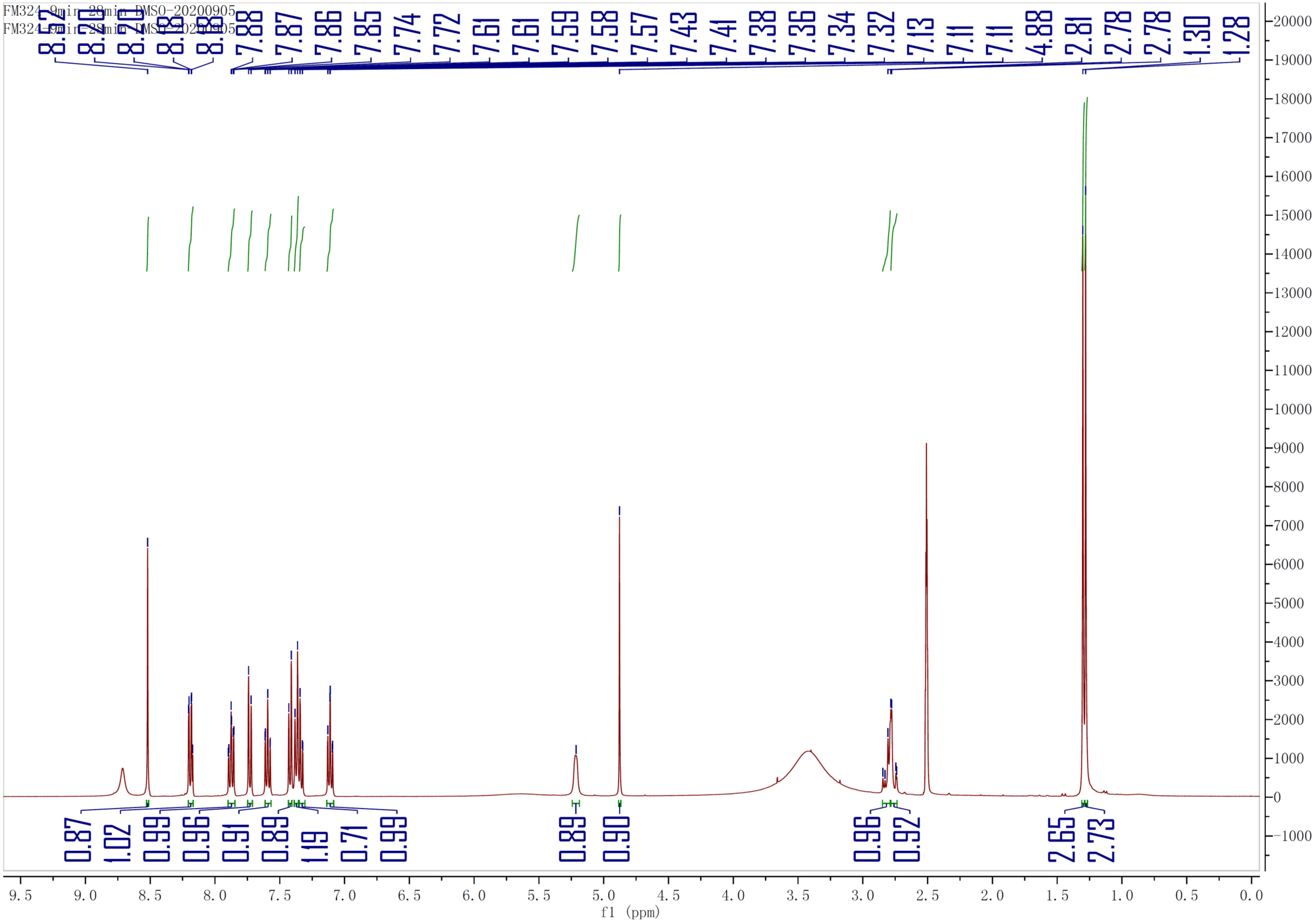

Figure S18. ^{13}C-NMR spectrum of compound **3** in DMSO-d_6

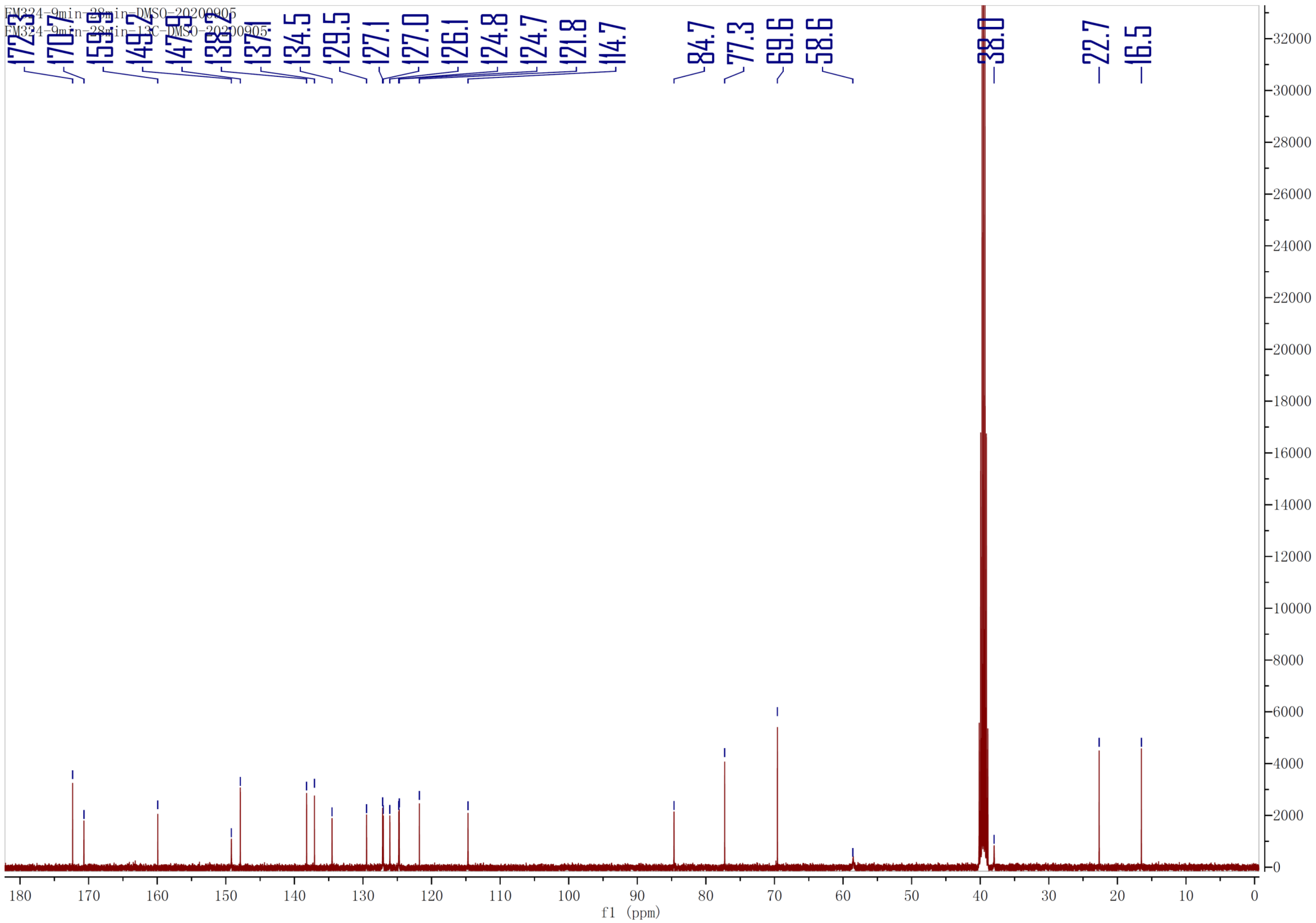

Figure S19. HSQC spectrum of compound **3** in DMSO-d_6

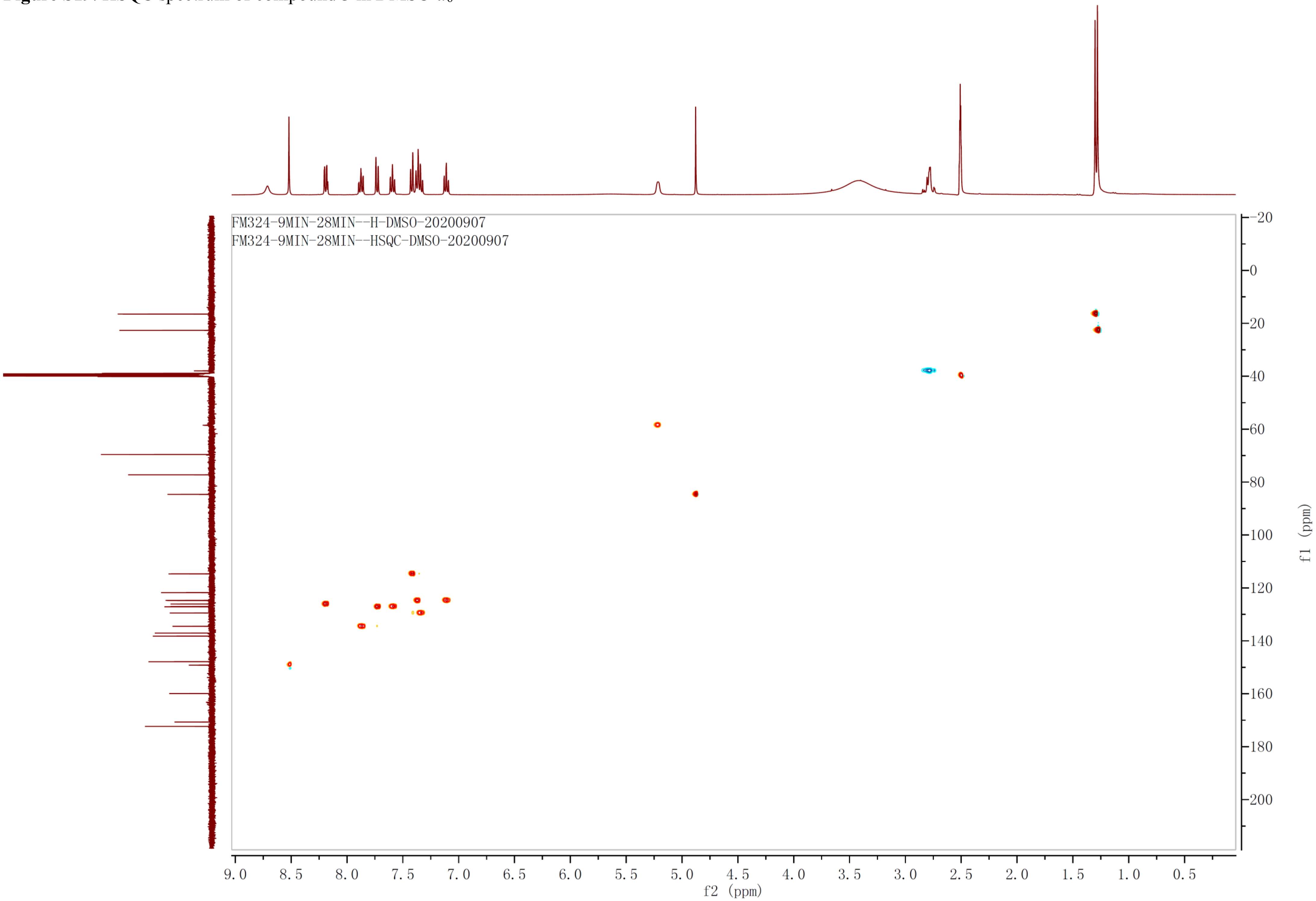

Figure S20. COSY spectrum of compound **3** in DMSO-d_6

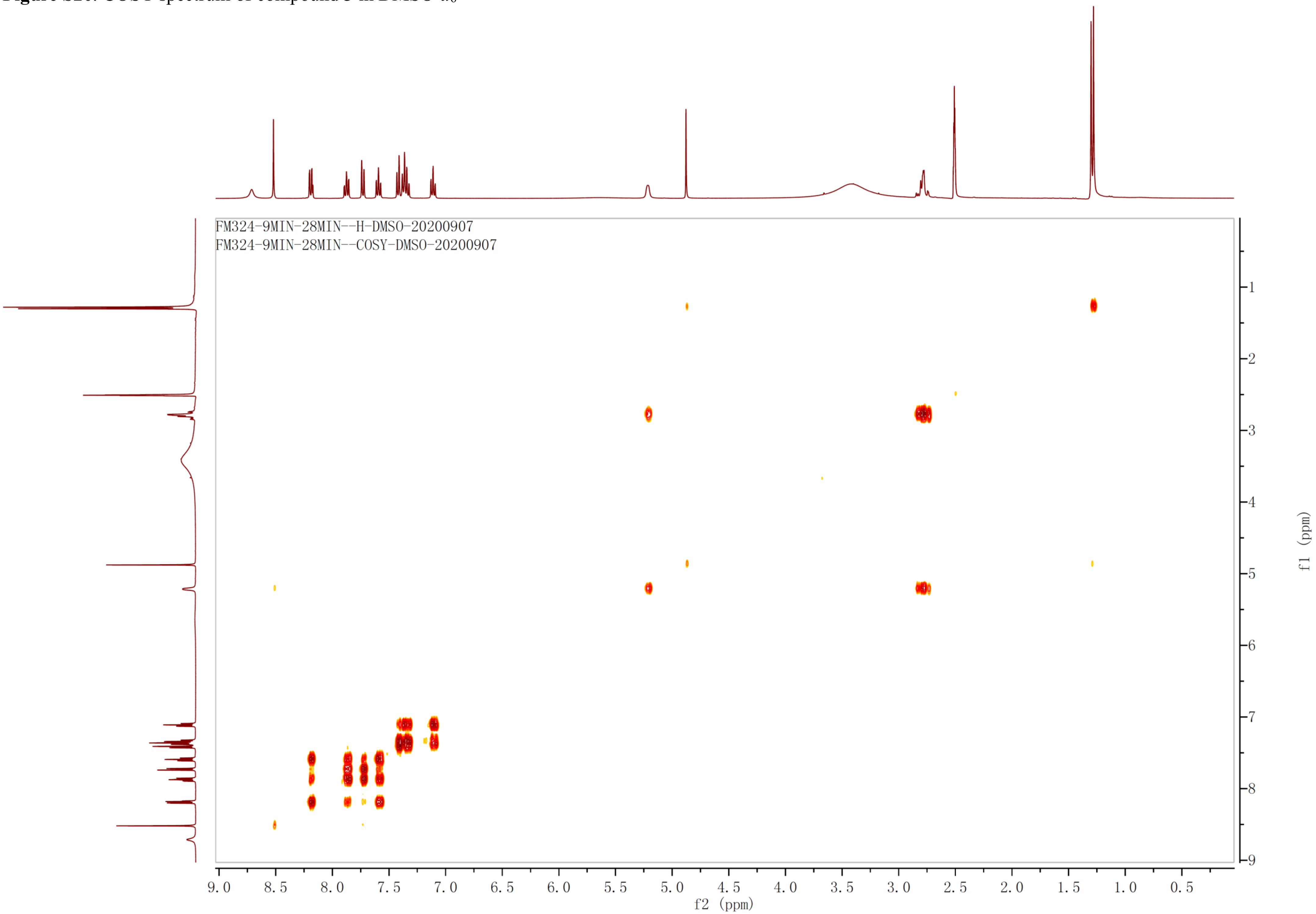

Figure S21. HMBC spectrum of compound **3** in DMSO-d_6

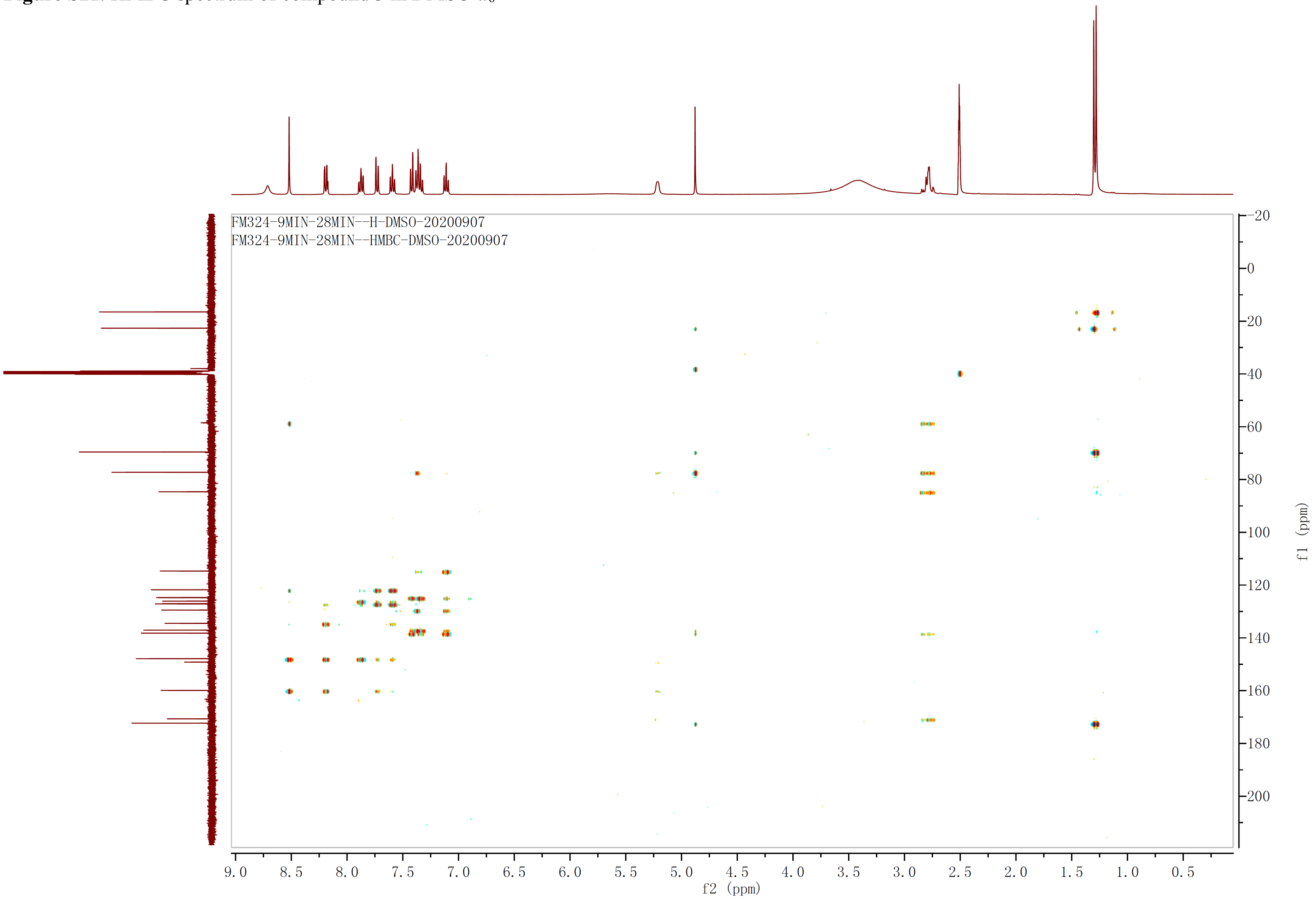

Figure S21. ROESY spectrum of compound **3** in DMSO-d_6

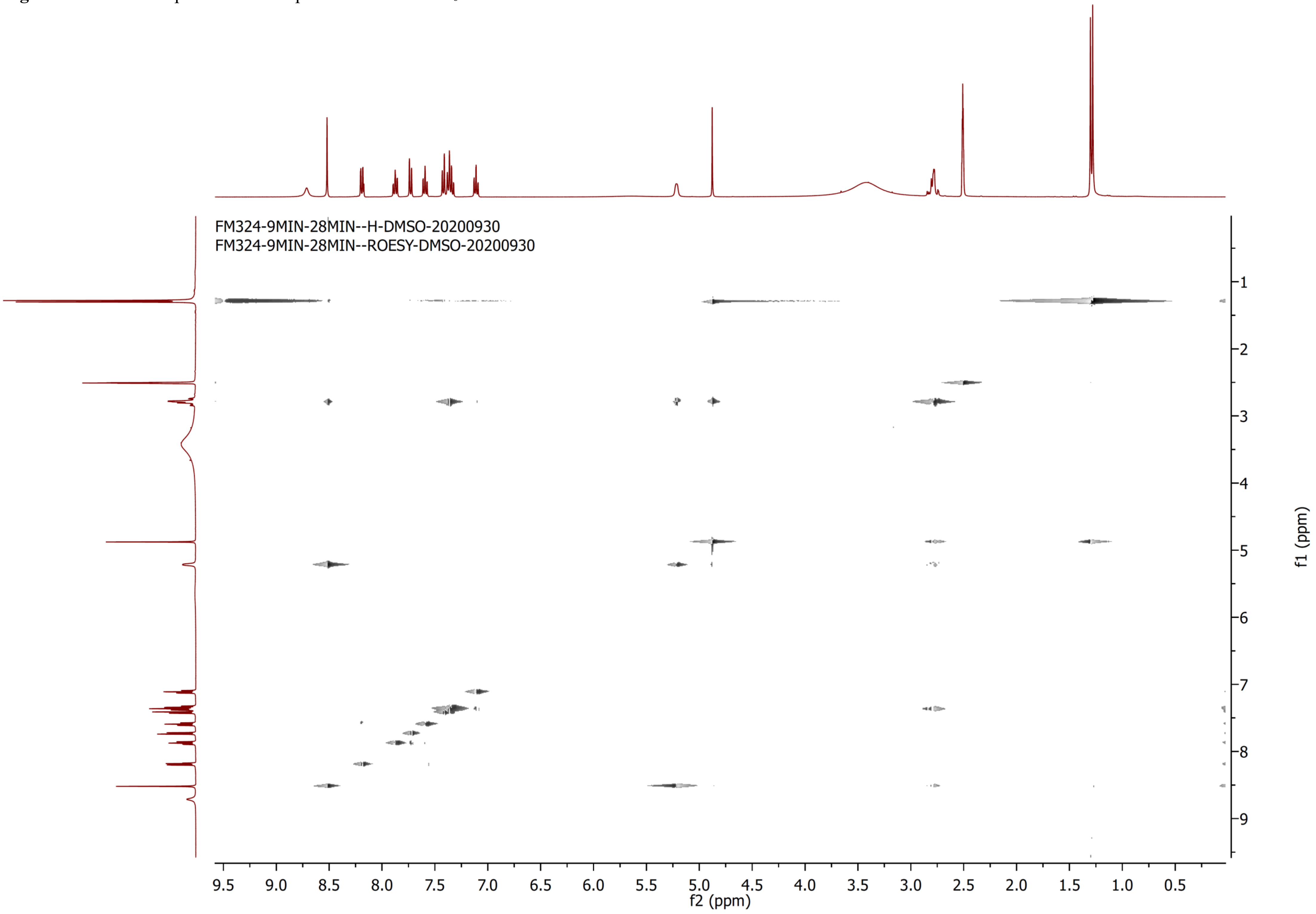

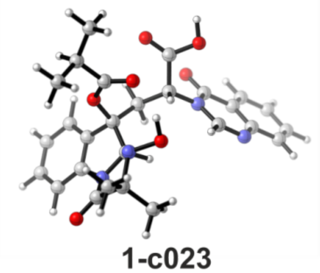

1-c023
G_{rel} = 0.0

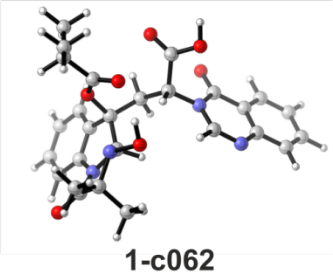

1-c062
G_{rel} = 0.2

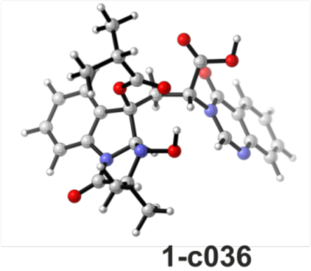

1-c036
G_{rel} = 0.5

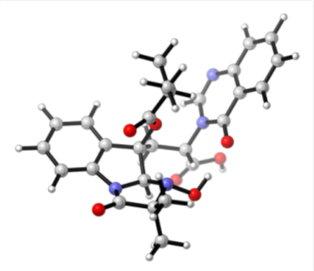

ent11-**1-c047**
G_{rel} = 0.0

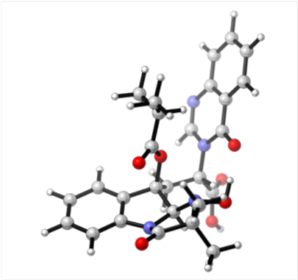

ent11-**1-c056**
G_{rel} = 0.2

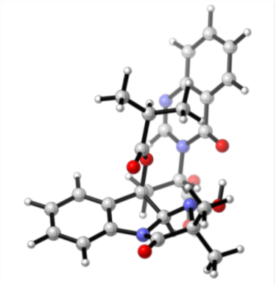

ent11-**1-c053**
G_{rel} = 1.3

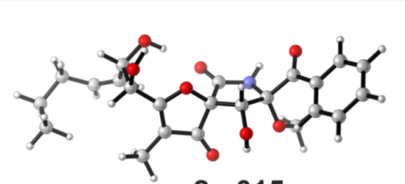

2-c015
G_{rel} = 0.0

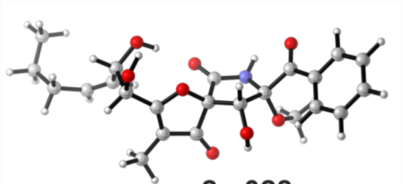

2-c028
G_{rel} = 0.6

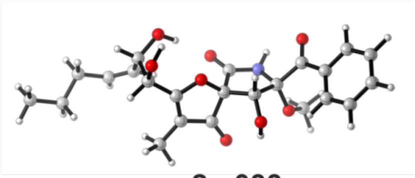

2-c033
G_{rel} = 0.6

Figure S23. B3LYP/6-31G* optimized geometries of the most stable conformers found for **1**, *11epi*-**1** and **2**, with relative Gibbs free energies (in kcal/mol) computed at the SMD/M06-2X/6-31G* level (solvent: methanol).

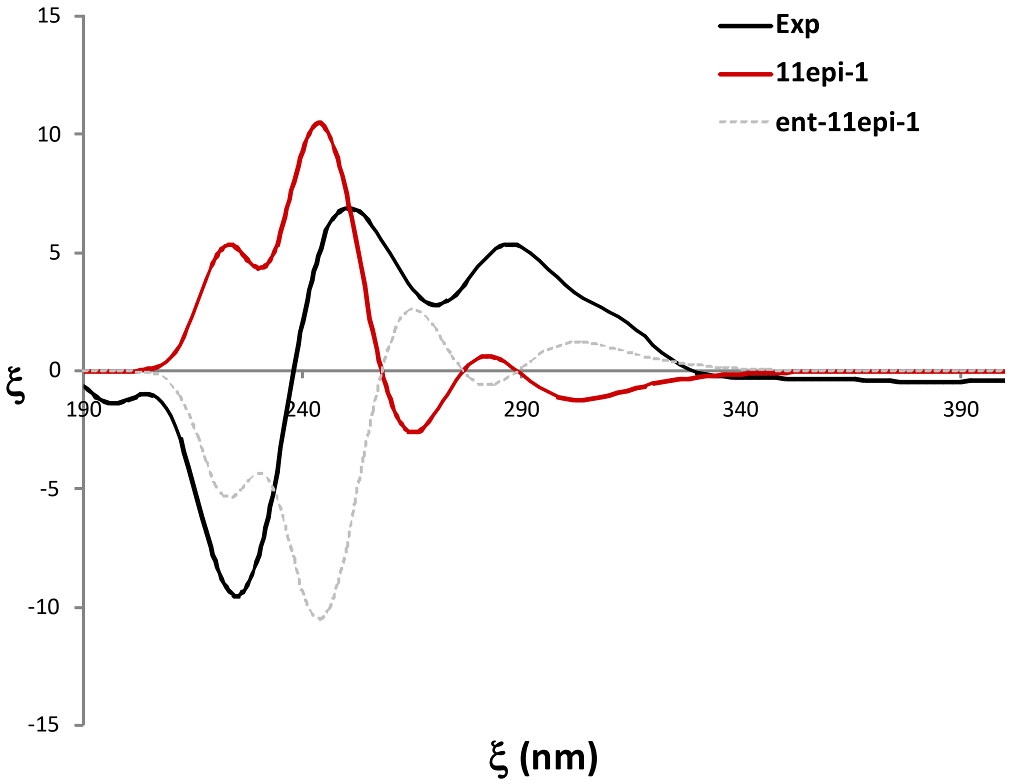

Figure S24. Experimental ECD of **1** and calculated ECD of *11epi*-**1**

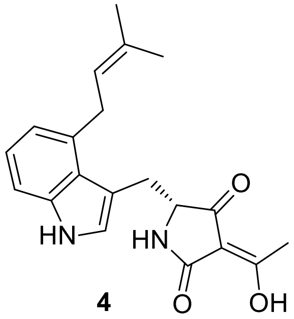

4

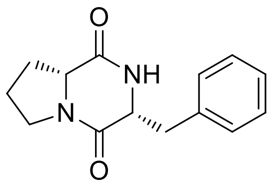

5

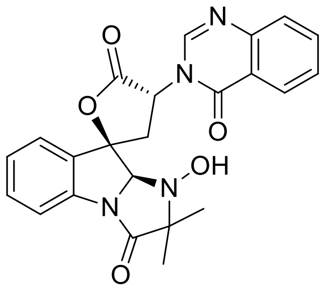

6

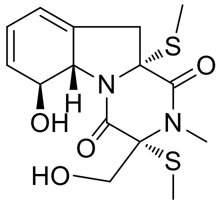

7

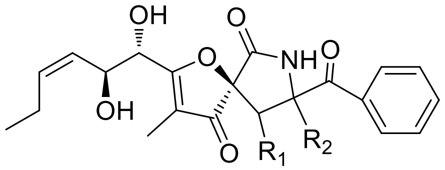

8: R_1 = ''''OH; R_2 = ''''OCH_3
9: R_1 = ◄OH; R_2 = ''''OCH_3
10: R_1 = ◄OH; R_2 = ◄OCH_3

Figure S25. Structures of compounds **4-10**

Cartesian coordinates of all compounds under study

11epi-1_c0002
B3LYP/6-31G* Geometry
C 2.360101 2.538478 1.671104
C 4.710708 1.611064 0.381443
C 3.532763 3.303357 1.678241
C 2.363661 1.306815 1.022163
C 3.542186 0.856259 0.404318
C 4.685114 2.849254 1.029624
N 3.331955 -0.424671 -0.124543
C 2.054349 -0.961274 0.325507
C 1.228448 0.307323 0.769416
N 1.606274 -1.740245 -0.822646
C 2.831233 -2.202797 -1.545438
C 3.794313 -1.030993 -1.270985
O 4.777827 -0.711803 -1.912022
C 3.442855 -3.494510 -0.966295
C 2.541254 -2.346107 -3.038272
O 0.835371 -2.853249 -0.365476
O 0.469213 0.704073 -0.412774
C -0.248723 1.853325 -0.432690
O -0.453417 2.552041 0.538975
C -0.752652 2.139770 -1.841656
C -1.821489 3.235997 -1.824017
C 0.439723 2.501024 -2.752586
C 0.339622 0.046152 1.993444
C -0.789537 -0.988961 1.804714
C -1.149052 -1.645805 3.144468
O -0.377436 -1.777954 4.066488
O -2.411924 -2.121799 3.162162
N -1.988986 -0.478072 1.115666
N -3.832033 1.026887 1.256662
C -3.656969 -0.539999 -0.617583
C -4.321244 0.502298 0.069238
C -2.419932 -1.095413 -0.069932
C -2.732420 0.530227 1.703918
O -1.796792 -2.024519 -0.587306
C -5.515390 1.022673 -0.465617
C -6.024018 0.513516 -1.650050
C -5.360326 -0.524978 -2.329947
C -4.185155 -1.049579 -1.816824
H 1.458201 2.914157 2.139739
H 5.595063 1.241171 -0.123608
H 3.541904 4.263861 2.185100
H 5.581877 3.462880 1.034873

H 2.194173 -1.602647 1.210890
H 4.385459 -3.709824 -1.479236
H 2.756354 -4.332118 -1.109398
H 3.649548 -3.402075 0.105412
H 2.092270 -1.429517 -3.431709
H 1.844061 -3.174140 -3.204308
H 3.467377 -2.545958 -3.585368
H -0.070897 -2.636736 -0.673387
H -1.186336 1.203574 -2.216753
H -1.408150 4.175352 -1.443624
H -2.666110 2.963128 -1.184262
H -2.198098 3.408416 -2.838058
H 1.177044 1.694166 -2.777884
H 0.086802 2.680957 -3.773615
H 0.935972 3.413244 -2.401525
H 0.984626 -0.338776 2.788364
H -0.069603 0.993553 2.348201
H -0.423704 -1.812151 1.182514
H -2.524955 -2.567347 4.023389
H -2.319572 0.917784 2.631367
H -6.012790 1.821606 0.074670
H -6.945617 0.919542 -2.058219
H -5.771205 -0.914560 -3.256715
H -3.653452 -1.851849 -2.317454
SCF Energy (B3LYP/6-31G*)= -1791.33199853

11epi-1_c0020
B3LYP/6-31G* Geometry
C 2.353793 2.580640 1.607835
C 4.691678 1.638319 0.306261
C 3.524748 3.347925 1.590102
C 2.352766 1.338773 0.978241
C 3.525430 0.881058 0.354426
C 4.670686 2.886348 0.935383
N 3.312886 -0.408383 -0.151893
C 2.043170 -0.942450 0.322829
C 1.217461 0.332180 0.752528
N 1.583341 -1.746053 -0.803416
C 2.799105 -2.205839 -1.543696
C 3.759704 -1.026264 -1.298838
O 4.731801 -0.709719 -1.958156
C 3.430873 -3.487155 -0.962856
C 2.483162 -2.366745 -3.029525
O 0.838013 -2.861345 -0.312511
O 0.442527 0.705440 -0.423922
C -0.276657 1.855154 -0.452645
O -0.460543 2.571492 0.509964

C -0.810558 2.113085 -1.855469
C -1.875615 3.213065 -1.837057
C 0.361262 2.449624 -2.801820
C 0.343107 0.089299 1.993533
C -0.788412 -0.960241 1.832133
C -1.196777 -1.548799 3.187003
O -2.297039 -1.497843 3.683728
O -0.145480 -2.165126 3.771914
N -1.985461 -0.469367 1.133913
N -3.825012 1.043840 1.214369
C -3.662276 -0.606529 -0.587500
C -4.320491 0.467180 0.055166
C -2.424652 -1.138298 -0.020858
C -2.727525 0.563115 1.685052
O -1.806166 -2.092696 -0.498615
C -5.515479 0.966281 -0.498708
C -6.030028 0.406070 -1.656904
C -5.371742 -0.663688 -2.292853
C -4.196221 -1.167847 -1.761341
H 1.455986 2.962880 2.078958
H 5.570857 1.262908 -0.203741
H 3.536999 4.316592 2.081105
H 5.565808 3.502189 0.920252
H 2.195190 -1.566890 1.218195
H 4.366645 -3.699504 -1.489377
H 2.748913 -4.331441 -1.086483
H 3.654041 -3.382605 0.104442
H 2.020578 -1.457483 -3.424211
H 1.788562 -3.200977 -3.173777
H 3.400374 -2.566305 -3.591568
H -0.079253 -2.662469 -0.600838
H -1.255965 1.170424 -2.199353
H -1.449585 4.160491 -1.492157
H -2.703178 2.959169 -1.168077
H -2.278398 3.360815 -2.844892
H 1.096432 1.640716 -2.825370
H -0.015249 2.606150 -3.818225
H 0.867199 3.368844 -2.484400
H 1.011816 -0.269946 2.781041
H -0.070533 1.040389 2.332400
H -0.410358 -1.797451 1.241262
H -0.471984 -2.529666 4.616432
H -2.315574 0.987283 2.593860
H -6.009224 1.789171 0.007934
H -6.952582 0.795565 -2.078915
H -5.787510 -1.093228 -3.199584
H -3.668273 -1.993050 -2.227497
SCF Energy (B3LYP/6-31G*)= -1791.33244423

11epi-1_c0045
B3LYP/6-31G* Geometry

C	-2.145088	1.173543	2.944467
C	-4.625577	0.543288	1.727541
C	-3.323463	1.147564	3.699578
C	-2.212157	0.891949	1.585321
C	-3.445607	0.586709	0.992839
C	-4.543127	0.828749	3.094077
N	-3.267027	0.330685	-0.376343
C	-1.903081	0.646213	-0.782252
C	-1.088987	0.790948	0.561839
N	-1.534374	-0.428000	-1.698728
C	-2.806410	-0.961819	-2.280709
C	-3.808352	-0.688525	-1.140467
O	-4.882051	-1.226015	-0.962123
C	-3.273637	-0.202811	-3.538809
C	-2.680527	-2.461224	-2.546392
O	-0.689856	0.112849	-2.721678
O	-0.175160	-0.310839	0.827856
C	-0.624334	-1.588629	0.983094
O	-1.783076	-1.925136	0.908719
C	0.532708	-2.540291	1.258759
C	0.172340	-3.472871	2.424466
C	0.843729	-3.333093	-0.026295
C	-0.185998	2.032030	0.552374
C	0.939262	2.024272	-0.507436
C	1.258128	3.448343	-0.981516
O	0.467703	4.363747	-0.965751
O	2.506294	3.555829	-1.483234
N	2.156063	1.305828	-0.095027
N	3.976121	1.270519	1.441879
C	3.801411	-0.434310	-0.309235
C	4.459739	0.136109	0.805277
C	2.566692	0.170478	-0.809226
C	2.891873	1.781053	0.972127
O	1.927048	-0.261482	-1.771142
C	5.644334	-0.458142	1.279986
C	6.153780	-1.584868	0.654313
C	5.499688	-2.146108	-0.458384
C	4.331879	-1.574832	-0.937579
H	-1.193595	1.396117	3.421261
H	-5.561606	0.277252	1.251373
H	-3.289022	1.371861	4.761711
H	-5.448316	0.803343	3.694659
H	-1.876671	1.614812	-1.304283
H	-4.260312	-0.573373	-3.833710

H -2.570256 -0.361346 -4.359540
H -3.351140 0.874980 -3.359707
H -2.363177 -2.980470 -1.638597
H -1.942485 -2.638403 -3.336248
H -3.644879 -2.868787 -2.864237
H   0.190007 -0.256844 -2.501974
H   1.409927 -1.940465 1.523584
H -0.737486 -4.035337 2.194306
H -0.002581 -2.911956 3.349308
H   0.986790 -4.182733 2.606073
H   1.101206 -2.664430 -0.853334
H   1.688327 -4.008807 0.148351
H -0.022506 -3.933859 -0.323086
H -0.809915 2.907882 0.358800
H   0.243072 2.164813 1.551336
H   0.587369 1.505858 -1.406208
H   2.592435 4.471427 -1.810947
H   2.490430 2.678645 1.437188
H   6.135690 -0.007719 2.136338
H   7.069044 -2.038693 1.024533
H   5.912320 -3.026804 -0.941691
H   3.809313 -1.984155 -1.795457
SCF Energy (B3LYP/6-31G*)= -1791.33226994

11epi-1_c0047
B3LYP/6-31G* Geometry
C -2.086467 0.688095 3.081946
C -4.597816 0.275568 1.836250
C -3.249661 0.554624 3.849360
C -2.183584 0.621811 1.697433
C -3.432478 0.422986 1.091881
C -4.484874 0.344369 3.228522
N -3.285458 0.381916 -0.304380
C -1.926246 0.744093 -0.686652
C -1.083083 0.677384 0.645936
N -1.588007 -0.189088 -1.755975
C -2.875891 -0.605738 -2.394146
C -3.854589 -0.501075 -1.206166
O -4.931748 -1.048790 -1.091400
C -3.348266 0.348565 -3.509199
C -2.777060 -2.045846 -2.895140
O -0.739879 0.477927 -2.697521
O -0.171359 -0.453177 0.702214
C -0.625721 -1.739921 0.669688
O -1.790454 -2.054070 0.604334
C   0.544527 -2.711946 0.764848
C   1.192350 -2.626510 2.162248
C   0.087678 -4.134978 0.431412

C -0.173785 1.902957 0.809969
C 0.928061 2.055167 -0.263145
C 1.231403 3.533838 -0.537233
O 0.439840 4.433170 -0.370869
O 2.465366 3.718684 -1.051443
N 2.155990 1.293511 0.018595
N 4.018424 1.064405 1.487486
C 3.793479 -0.399790 -0.463459
C 4.485402 0.025308 0.694222
C 2.543886 0.258508 -0.845657
C 2.921044 1.627581 1.118624
O 1.876249 -0.053013 -1.833267
C 5.685091 -0.619061 1.050901
C 6.175180 -1.653671 0.269569
C 5.485271 -2.072058 -0.883582
C 4.302276 -1.449423 -1.248029
H -1.123533 0.826530 3.567390
H -5.546254 0.095020 1.344754
H -3.191015 0.610377 4.932446
H -5.378123 0.233630 3.837241
H -1.900002 1.778569 -1.062328
H -4.344299 0.042030 -3.843469
H -2.658519 0.312158 -4.355667
H -3.407056 1.385566 -3.161417
H -2.454826 -2.706917 -2.086478
H -2.052475 -2.104445 -3.714660
H -3.751448 -2.385827 -3.258557
H 0.134413 0.065913 -2.538296
H 1.285063 -2.382907 0.024844
H 0.476866 -2.918233 2.940125
H 1.544646 -1.614364 2.380951
H 2.048630 -3.306911 2.220483
H -0.341031 -4.190063 -0.573851
H 0.937528 -4.824451 0.482151
H -0.677997 -4.473626 1.136160
H -0.797685 2.799127 0.770361
H 0.278881 1.877273 1.807133
H 0.559549 1.667262 -1.219556
H 2.540732 4.671952 -1.248194
H 2.532061 2.454514 1.708761
H 6.202980 -0.280319 1.942365
H 7.102446 -2.146324 0.549226
H 5.882021 -2.882699 -1.487649
H 3.750935 -1.748925 -2.133149
SCF Energy (B3LYP/6-31G*)= -1791.33195089

11epi-1_c0053

B3LYP/6-31G* Geometry

```
C  -2.116413  1.200653  2.939328
C  -4.599952  0.529379  1.750683
C  -3.289348  1.178264  3.703020
C  -2.190829  0.895461  1.585591
C  -3.425593  0.569934  1.006746
C  -4.510433  0.839116  3.111502
N  -3.254346  0.290754 -0.358767
C  -1.895931  0.610015 -0.780829
C  -1.074226  0.787147  0.555607
N  -1.522350 -0.478728 -1.678061
C  -2.792873 -1.040868 -2.236439
C  -3.790045 -0.751543 -1.096537
O  -4.857184 -1.294822 -0.898228
C  -3.278182 -0.317929 -3.508998
C  -2.651738 -2.544703 -2.467480
O  -0.696675  0.050704 -2.721308
O  -0.144075 -0.294746  0.832653
C  -0.577025 -1.575954  1.013900
O  -1.733256 -1.924240  0.960415
C   0.594444 -2.509751  1.287254
C   0.255533 -3.440338  2.460834
C   0.907501 -3.305697  0.004368
C  -0.188656  2.042511  0.518877
C   0.936733  2.027629 -0.553849
C   1.290619  3.444470 -1.015914
O   2.370441  3.978773 -0.923569
O   0.211155  4.039592 -1.570373
N   2.154613  1.324712 -0.133842
N   3.967248  1.290047  1.411384
C   3.810549 -0.405835 -0.349370
C   4.460749  0.163906  0.770027
C   2.572888  0.191575 -0.847684
C   2.884906  1.802787  0.938451
O   1.935796 -0.244964 -1.810622
C   5.647755 -0.425837  1.245561
C   6.166800 -1.545475  0.615656
C   5.520348 -2.105742 -0.502381
C   4.350375 -1.540063 -0.982004
H  -1.163233  1.437470  3.405897
H  -5.536848  0.246526  1.286057
H  -3.249331  1.420234  4.761052
H  -5.411132  0.816111  3.718874
H  -1.880023  1.567408 -1.323220
H  -4.262877 -0.706635 -3.786617
H  -2.579224 -0.488719 -4.330950
H  -3.366083  0.763047 -3.355664
H  -2.325218 -3.039304 -1.549270
```

H -1.914666 -2.731745 -3.255881
H -3.612640 -2.970194 -2.772033
H 0.194167 -0.290913 -2.498168
H 1.465131 -1.896198 1.541768
H -0.647616 -4.017319 2.240572
H 0.079148 -2.876571 3.383645
H 1.081488 -4.137159 2.640656
H 1.145857 -2.638792 -0.829691
H 1.765574 -3.964830 0.176104
H 0.049437 -3.923872 -0.280169
H -0.840167 2.897842 0.323306
H 0.248780 2.197204 1.510597
H 0.573080 1.503047 -1.441149
H 0.498062 4.926897 -1.858647
H 2.482837 2.697735 1.401858
H 6.133500 0.023966 2.105417
H 7.084231 -1.994700 0.986268
H 5.940945 -2.980790 -0.989100
H 3.833293 -1.948279 -1.843755
SCF Energy (B3LYP/6-31G*)= -1791.33280638

11epi-1_c0056
B3LYP/6-31G* Geometry
C -2.054359 0.732921 3.074445
C -4.568822 0.260517 1.856419
C -3.210335 0.602079 3.853103
C -2.160682 0.634641 1.692387
C -3.410813 0.406088 1.100138
C -4.447026 0.362292 3.245945
N -3.273075 0.333624 -0.295728
C -1.920976 0.703230 -0.697193
C -1.068463 0.680179 0.631812
N -1.576383 -0.251494 -1.745437
C -2.862410 -0.709060 -2.358855
C -3.833760 -0.585567 -1.167372
O -4.901215 -1.146077 -1.027698
C -3.361083 0.203781 -3.497169
C -2.742846 -2.161235 -2.818846
O -0.753603 0.406699 -2.714256
O -0.136607 -0.429305 0.709851
C -0.569507 -1.724803 0.714807
O -1.729664 -2.057729 0.665686
C 0.617436 -2.674006 0.827187
C 1.291611 -2.522666 2.206455
C 0.177014 -4.115999 0.559494
C -0.180168 1.927431 0.762450
C 0.922995 2.068595 -0.323751

C   1.264621 3.537476 -0.590788
O   2.345168 4.059101 -0.447826
O   0.172122 4.199175 -1.033140
N   2.149982 1.318846 -0.029419
N   3.999132 1.081150 1.454328
C   3.793599 -0.370633 -0.507361
C   4.475308 0.050240 0.657665
C   2.544615 0.284657 -0.892368
C   2.906804 1.651256 1.079176
O   1.881468 -0.028488 -1.883677
C   5.674391 -0.593648 1.018827
C   6.172979 -1.621836 0.234855
C   5.492428 -2.036104 -0.925714
C   4.310310 -1.415069 -1.294443
H -1.089523 0.892386 3.549711
H -5.518086 0.055617 1.376233
H -3.144736 0.681922 4.934264
H -5.334394 0.253303 3.863501
H -1.908503 1.727578 -1.099622
H -4.354677 -0.129302 -3.812797
H -2.678232 0.154313 -4.348457
H -3.434081 1.249344 -3.179003
H -2.403254 -2.793145 -1.994216
H -2.023215 -2.230786 -3.641793
H -3.713851 -2.528060 -3.164824
H   0.134234 0.026246 -2.548954
H   1.338496 -2.363830 0.060070
H   0.595464 -2.789940 3.010300
H   1.636999 -1.498824 2.375702
H   2.156688 -3.190756 2.274854
H -0.270591 -4.217781 -0.433755
H   1.038931 -4.789380 0.620244
H -0.568806 -4.438336 1.292691
H -0.833460 2.801956 0.709932
H   0.279254 1.934371 1.756382
H   0.542814 1.670621 -1.268445
H   0.451065 5.119361 -1.200929
H   2.518095 2.476560 1.666736
H   6.185245 -0.258029 1.915503
H   7.100121 -2.112953 0.517801
H   5.896145 -2.841858 -1.531729
H   3.765745 -1.711001 -2.184963
SCF Energy (B3LYP/6-31G*)= -1791.33248473

1_c0023
B3LYP/6-31G* Geometry
C   1.657843 -0.478847 3.329765
C   3.008092 -2.773166 2.352549

C	2.318716	-1.346861	4.206041
C	1.666558	-0.765484	1.969506
C	2.333421	-1.902961	1.500747
C	2.989605	-2.473186	3.717663
N	2.200725	-1.979685	0.105682
C	1.283149	-0.956787	-0.389942
C	1.105298	0.056780	0.808307
N	1.945219	-0.519375	-1.613870
C	2.671301	-1.703892	-2.164168
C	3.102301	-2.392096	-0.854211
O	4.034749	-3.154046	-0.688107
C	1.772843	-2.663145	-2.970944
C	3.879138	-1.253247	-2.982158
O	0.973881	-0.042215	-2.548630
O	2.018395	1.203188	0.755040
C	2.034733	2.171392	-0.176213
O	1.296617	2.251645	-1.145077
C	3.135359	3.177359	0.126824
C	4.450316	2.683989	-0.513477
C	2.738088	4.572562	-0.368095
C	-0.352237	0.469568	1.090574
C	-1.177842	1.095767	-0.059792
C	-1.423389	2.590717	0.179538
O	-0.780571	3.282247	0.934899
O	-2.387219	3.072699	-0.631017
N	-2.436344	0.378533	-0.316633
N	-3.684108	-0.959619	-1.832413
C	-4.660016	-0.282625	0.316532
C	-4.733224	-0.963282	-0.922078
C	-3.443696	0.452956	0.662220
C	-2.622934	-0.306451	-1.500992
O	-3.252515	1.093547	1.692161
C	-5.910281	-1.666520	-1.243410
C	-6.973771	-1.685413	-0.354571
C	-6.895437	-1.005718	0.875277
C	-5.745415	-0.307873	1.208057
H	1.157898	0.408115	3.708138
H	3.531697	-3.635924	1.958679
H	2.315096	-1.140439	5.272185
H	3.505049	-3.132413	4.410626
H	0.298676	-1.399501	-0.607296
H	2.348848	-3.552104	-3.246109
H	1.419563	-2.171240	-3.880413
H	0.899726	-2.989128	-2.395481
H	4.503714	-0.567291	-2.402665
H	3.542880	-0.740072	-3.888994
H	4.484219	-2.118804	-3.267289

H 1.090740 0.924483 -2.448629
H 3.267755 3.191647 1.213945
H 4.352217 2.622253 -1.603094
H 4.731275 1.696240 -0.134790
H 5.260871 3.383614 -0.283340
H 1.806366 4.905943 0.099911
H 3.525672 5.294254 -0.126002
H 2.589355 4.570833 -1.452176
H -0.866847 -0.436670 1.420762
H -0.352254 1.155167 1.939764
H -0.615188 1.047532 -0.991941
H -2.458134 4.025251 -0.432891
H -1.772776 -0.282658 -2.180322
H -5.953085 -2.183503 -2.196708
H -7.878439 -2.230649 -0.610234
H -7.736882 -1.028785 1.561692
H -5.654232 0.227126 2.147845
SCF Energy (B3LYP/6-31G*)= -1791.32909567

1_c0029
B3LYP/6-31G* Geometry
C 0.690419 2.446802 -1.630772
C 3.497778 2.337937 -2.006841
C 1.316093 3.228383 -2.609384
C 1.473938 1.634457 -0.812417
C 2.859306 1.579066 -1.030629
C 2.700586 3.174807 -2.791515
N 3.445045 0.642673 -0.164291
C 2.477934 0.159369 0.809444
C 1.060157 0.618274 0.255392
N 2.777281 -1.266796 0.895715
C 4.233866 -1.421599 0.595382
C 4.442266 -0.279558 -0.416722
O 5.312353 -0.188142 -1.260691
C 5.144774 -1.179707 1.816986
C 4.509031 -2.784224 -0.036550
O 2.497002 -1.730248 2.217508
O 0.392260 -0.435615 -0.504803
C -0.025201 -1.596092 0.039091
O -0.038387 -1.843420 1.234473
C -0.529366 -2.542059 -1.042663
C 0.638333 -2.980040 -1.950852
C -1.264484 -3.734981 -0.424730
C 0.228756 1.149795 1.441385
C -1.165856 1.748458 1.184868
C -1.594551 2.593477 2.394420
O -0.824516 3.152113 3.141513
O -2.935717 2.695725 2.502020

N -2.214200 0.794832 0.790155
N -3.579541 -1.037243 1.456919
C -3.959249 0.062752 -0.701921
C -4.271487 -0.928108 0.257581
C -2.885069 1.019305 -0.431513
C -2.622329 -0.199357 1.658678
O -2.564832 1.945053 -1.172324
C -5.317570 -1.831802 -0.009868
C -6.023590 -1.744258 -1.199697
C -5.709365 -0.756234 -2.151175
C -4.685054 0.143470 -1.902629
H -0.391991 2.449581 -1.556243
H 4.567498 2.253868 -2.155081
H 0.711691 3.870941 -3.242917
H 3.167083 3.782377 -3.562197
H 2.631690 0.639922 1.786139
H 6.190480 -1.198650 1.494301
H 4.985869 -1.959635 2.565180
H 4.949806 -0.210212 2.287389
H 3.870435 -2.941313 -0.910444
H 4.309397 -3.576991 0.691764
H 5.553749 -2.847708 -0.354617
H 1.598469 -2.099589 2.103847
H -1.227779 -1.957470 -1.656611
H 1.132804 -2.117666 -2.405504
H 0.262527 -3.627101 -2.750448
H 1.383458 -3.543769 -1.378797
H -2.086295 -3.409233 0.219309
H -1.673257 -4.370597 -1.217398
H -0.584115 -4.339700 0.183718
H 0.165990 0.359405 2.193556
H 0.810833 1.958792 1.891382
H -1.122190 2.459111 0.354730
H -3.105507 3.287945 3.259334
H -2.056754 -0.259787 2.584178
H -5.547206 -2.584926 0.737084
H -6.829379 -2.445457 -1.399594
H -6.271349 -0.699057 -3.078793
H -4.421739 0.918934 -2.614415
SCF Energy (B3LYP/6-31G*)= -1791.32731530

1_c0036
B3LYP/6-31G* Geometry
C 1.775842 0.599432 3.164033
C 3.047881 -1.924146 2.926021
C 2.459575 0.038278 4.248403
C 1.723238 -0.106618 1.967483

C	2.351725	-1.352656	1.864550
C	3.091572	-1.203583	4.123033
N	2.159543	-1.867365	0.572702
C	1.237386	-1.031378	-0.191938
C	1.125939	0.316116	0.624646
N	1.857156	-1.019048	-1.512749
C	2.551545	-2.331042	-1.682382
C	3.021299	-2.580655	-0.235983
O	3.950483	-3.271644	0.133393
C	1.616481	-3.477342	-2.118291
C	3.735550	-2.184931	-2.635404
O	0.857273	-0.841733	-2.519197
O	2.059638	1.349695	0.167711
C	2.044490	1.974830	-1.021719
O	1.249706	1.776176	-1.926720
C	3.149209	3.018532	-1.094647
C	2.891219	4.140503	-0.070848
C	4.538130	2.382674	-0.911977
C	-0.309198	0.845355	0.811385
C	-1.183603	1.070191	-0.446454
C	-1.448992	2.562433	-0.681351
O	-0.792063	3.462344	-0.211365
O	-2.451104	2.757009	-1.562303
N	-2.435791	0.298695	-0.420847
N	-3.708133	-1.453383	-1.398686
C	-4.626911	-0.145425	0.465264
C	-4.727658	-1.179101	-0.496167
C	-3.412802	0.668761	0.520873
C	-2.647991	-0.723035	-1.325414
O	-3.200007	1.600558	1.291855
C	-5.902355	-1.954577	-0.540561
C	-6.936697	-1.701753	0.346912
C	-6.831044	-0.671792	1.300172
C	-5.682976	0.102080	1.358030
H	1.304362	1.573595	3.256401
H	3.540955	-2.881884	2.811059
H	2.503838	0.573988	5.191987
H	3.625286	-1.621410	4.972172
H	0.238845	-1.493644	-0.225008
H	2.173819	-4.419216	-2.114670
H	1.237194	-3.290815	-3.125912
H	0.760182	-3.586685	-1.444082
H	4.387886	-1.366344	-2.317249
H	3.374885	-1.972366	-3.647070
H	4.320726	-3.108960	-2.653222
H	0.986442	0.104142	-2.735656
H	3.071228	3.432810	-2.105299
H	2.990331	3.760882	0.950934

H 1.886326 4.559711 -0.183807
H 3.621667 4.944771 -0.211549
H 4.713555 1.584937 -1.642263
H 5.315089 3.143032 -1.047383
H 4.643775 1.957208 0.090467
H -0.820836 0.124918 1.454733
H -0.253480 1.778939 1.374437
H -0.649000 0.738074 -1.336132
H -2.531780 3.722765 -1.673743
H -1.819922 -0.907697 -2.007278
H -5.966659 -2.743443 -1.283156
H -7.839685 -2.305101 0.305921
H -7.649825 -0.484765 1.988895
H -5.571175 0.904132 2.080529
SCF Energy (B3LYP/6-31G*)= -1791.32872869

1_c0062
B3LYP/6-31G* Geometry
C 1.711344 -0.170062 3.309861
C 2.991826 -2.588569 2.565654
C 2.359361 -0.959740 4.266303
C 1.698577 -0.596146 1.986802
C 2.330328 -1.793901 1.633605
C 2.996114 -2.148009 3.892322
N 2.179836 -2.012011 0.255068
C 1.280289 -1.024225 -0.335217
C 1.148983 0.115930 0.749713
N 1.937643 -0.730410 -1.604087
C 2.639248 -1.976778 -2.034570
C 3.063718 -2.539910 -0.663995
O 3.979676 -3.303155 -0.427543
C 1.721271 -2.994793 -2.741181
C 3.853512 -1.632949 -2.894393
O 0.967814 -0.331439 -2.576071
O 2.109011 1.206784 0.555075
C 2.127228 2.075737 -0.470342
O 1.349895 2.090567 -1.411064
C 3.239301 3.096265 -0.273169
C 3.554431 3.821318 -1.584808
C 2.833279 4.075865 0.849748
C -0.288359 0.612131 1.001075
C -1.129662 1.091162 -0.207242
C -1.381187 2.603197 -0.145638
O -0.739999 3.383650 0.518812
O -2.352333 2.980277 -1.002198
N -2.388722 0.345203 -0.361651
N -3.653644 -1.156979 -1.699132

C -4.603772 -0.239137 0.370632
C -4.691081 -1.056918 -0.781127
C -3.384950 0.532631 0.613610
C -2.589423 -0.470115 -1.457789
O -3.183241 1.289774 1.559012
C -5.871242 -1.791882 -1.005453
C -6.923583 -1.708699 -0.107123
C -6.831135 -0.893081 1.036070
C -5.677955 -0.161986 1.272707
H 1.236683 0.763505 3.598380
H 3.488753 -3.501022 2.258905
H 2.372078 -0.644132 5.305312
H 3.501975 -2.745156 4.645948
H 0.282558 -1.462085 -0.494418
H 2.281922 -3.915331 -2.930816
H 1.371048 -2.587907 -3.692937
H 0.845784 -3.249488 -2.134197
H 4.489271 -0.900887 -2.388071
H 3.526182 -1.211304 -3.850346
H 4.444195 -2.533291 -3.086484
H 1.107532 0.637276 -2.581491
H 4.122026 2.539626 0.065062
H 2.682391 4.377456 -1.942098
H 3.847812 3.118336 -2.371160
H 4.377352 4.527715 -1.432554
H 2.675948 3.549049 1.794932
H 3.624200 4.819446 0.995460
H 1.906513 4.599755 0.593463
H -0.819372 -0.217578 1.474639
H -0.244604 1.414520 1.739952
H -0.578110 0.932686 -1.133778
H -2.429010 3.949003 -0.917103
H -1.748269 -0.522714 -2.146486
H -5.925436 -2.414365 -1.892825
H -7.830747 -2.279257 -0.287689
H -7.664124 -0.837517 1.730867
H -5.575690 0.477354 2.143628
SCF Energy (B3LYP/6-31G*)= -1791.32912769

1_c0063
B3LYP/6-31G* Geometry
C 1.474862 1.795332 2.599623
C 3.787328 0.211771 3.045736
C 2.352061 2.084538 3.651634
C 1.751925 0.710250 1.772805
C 2.891737 -0.073514 2.019994
C 3.498227 1.310981 3.859402
N 2.932335 -1.140728 1.111086

```
C   1.686784 -1.219557  0.363387
C   1.045293  0.213175  0.506875
N   2.125219 -1.670874 -0.950406
C   3.360639 -2.484769 -0.747232
C   3.978092 -1.772730  0.474820
O   5.141394 -1.780778  0.829196
C   3.082413 -3.956525 -0.379078
C   4.270293 -2.381218 -1.969911
O   1.093740 -2.444859 -1.566807
O   1.497176  0.956232 -0.659977
C   1.236528  2.284285 -0.807709
O   0.571157  2.940400 -0.035172
C   1.911305  2.807328 -2.066224
C   1.229215  4.089482 -2.551173
C   3.415243  3.012171 -1.785499
C  -0.489601  0.184510  0.598671
C  -1.201616 -0.491036 -0.600839
C  -0.955007  0.172416 -1.982200
O  -0.335269 -0.414804 -2.850241
O  -1.423670  1.397519 -2.176273
N  -2.650069 -0.686328 -0.314219
N  -4.321634 -2.340894  0.088690
C  -4.849975  0.034176  0.346758
C  -5.218061 -1.330681  0.401539
C  -3.497048  0.395831 -0.054547
C  -3.128162 -1.988908 -0.237331
O  -3.098220  1.565987 -0.166093
C  -6.529617 -1.672469  0.781279
C  -7.439041 -0.675807  1.096782
C  -7.069103  0.681833  1.039651
C  -5.784290  1.036683  0.664575
H   0.612369  2.424302  2.414182
H   4.668816 -0.400934  3.192833
H   2.144583  2.927348  4.304445
H   4.174921  1.560546  4.672130
H   1.003687 -1.948929  0.832180
H   4.027940 -4.456651 -0.147473
H   2.602259 -4.468403 -1.216489
H   2.428403 -4.042616  0.495778
H   4.445335 -1.333589 -2.230453
H   3.801504 -2.878878 -2.825244
H   5.233437 -2.857798 -1.764625
H   0.750313 -1.833624 -2.256167
H   1.810016  2.022306 -2.824806
H   1.278755  4.870796 -1.786348
H   0.175141  3.907940 -2.782170
H   1.722690  4.457234 -3.457335
```

H 3.888524 2.082024 -1.456663
H 3.920496 3.352064 -2.695872
H 3.567903 3.771612 -1.009855
H -0.754378 -0.394936 1.491310
H -0.860934 1.197822 0.744018
H -0.796439 -1.496213 -0.717506
H -1.970169 1.683520 -1.390932
H -2.394738 -2.753512 -0.481878
H -6.796137 -2.723670 0.816855
H -8.450296 -0.943976 1.389904
H -7.794607 1.450708 1.287886
H -5.473365 2.074405 0.608435
SCF Energy (B3LYP/6-31G*)= -1791.33437468

1_c0085
B3LYP/6-31G* Geometry
C 1.597272 -0.601493 3.332302
C 2.859350 -2.902007 2.257211
C 2.222001 -1.531627 4.170552
C 1.598475 -0.828386 1.960827
C 2.220938 -1.970126 1.443562
C 2.850016 -2.660727 3.634024
N 2.086675 -1.982250 0.046503
C 1.209517 -0.904958 -0.404546
C 1.075533 0.065065 0.834927
N 1.889835 -0.441695 -1.610719
C 2.568870 -1.631414 -2.210486
C 2.972570 -2.388718 -0.930444
O 3.875637 -3.191160 -0.796462
C 1.632433 -2.520341 -3.053693
C 3.793240 -1.197291 -3.012646
O 0.939849 0.110001 -2.525861
O 2.054285 1.158190 0.814979
C 2.128059 2.139106 -0.100596
O 1.338953 2.330557 -1.011374
C 3.356094 3.002629 0.154813
C 4.614117 2.251341 -0.331436
C 3.207506 4.373307 -0.511436
C -0.361342 0.536657 1.135133
C -1.179366 1.188303 -0.011103
C -1.533601 2.670485 0.183858
O -2.396997 3.238373 -0.440218
O -0.728416 3.297664 1.069934
N -2.419109 0.455574 -0.303876
N -3.671728 -0.774526 -1.903544
C -4.638140 -0.253002 0.292622
C -4.714903 -0.847183 -0.989465
C -3.423126 0.462043 0.681679

C -2.612447 -0.138683 -1.534544
O -3.227131 1.028162 1.753856
C -5.890664 -1.532320 -1.352204
C -6.949502 -1.616647 -0.461678
C -6.867986 -1.021813 0.811185
C -5.719156 -0.343334 1.185226
H 1.129433 0.285656 3.749378
H 3.348931 -3.767195 1.826642
H 2.223215 -1.372190 5.244721
H 3.337673 -3.369087 4.297973
H 0.208585 -1.298460 -0.638427
H 2.173162 -3.418846 -3.366629
H 1.298035 -1.977876 -3.941248
H 0.747578 -2.835695 -2.490447
H 4.448871 -0.565055 -2.406880
H 3.477810 -0.632279 -3.895813
H 4.359779 -2.075097 -3.336770
H 1.095227 1.067656 -2.398468
H 3.436144 3.123186 1.241960
H 4.565079 2.069679 -1.410988
H 4.721463 1.288515 0.176075
H 5.507033 2.851742 -0.127780
H 2.317868 4.898257 -0.149020
H 4.084720 4.992358 -0.295500
H 3.113670 4.271153 -1.596786
H -0.905820 -0.350215 1.468268
H -0.326835 1.205417 1.995743
H -0.595788 1.167443 -0.930968
H -1.019004 4.229176 1.074680
H -1.768209 -0.059870 -2.216609
H -5.936683 -1.982083 -2.338857
H -7.853452 -2.146663 -0.749941
H -7.706282 -1.094561 1.498016
H -5.625896 0.127732 2.158422
SCF Energy (B3LYP/6-31G*)= -1791.32593082

2_c0015
B3LYP/6-31G* Geometry
O -0.744771 -0.497368 -0.864057
C 0.131480 0.263267 -0.029626
C -0.558611 1.640370 0.102133
C -1.885445 1.452220 -0.451514
C -1.921984 0.186626 -0.953581
C 0.206518 -0.491988 1.308256
C 1.597544 0.319821 -0.516300
C 2.441854 0.158046 0.818141
N 1.487908 -0.406259 1.750590

O	-0.713320	-1.098866	1.835520
C	3.668421	-0.815395	0.762687
O	3.651102	-1.781678	1.515944
C	4.833276	-0.552906	-0.123472
C	7.105436	-0.211104	-1.724546
C	5.894307	-1.477573	-0.065576
C	4.925004	0.547080	-0.993621
C	6.058448	0.709002	-1.790175
C	7.021420	-1.307563	-0.859474
O	2.871097	1.461368	1.156839
C	3.365550	1.640074	2.481319
O	1.913952	1.468378	-1.243554
O	-0.030453	2.638003	0.576953
C	-2.940666	2.515414	-0.450848
C	-3.059976	-0.642678	-1.479462
C	-3.694442	-1.497295	-0.324011
C	-4.196357	-0.700293	0.849420
O	-2.638132	-1.516921	-2.503981
O	-2.752678	-2.494790	0.064157
C	-5.472867	-0.478807	1.189216
C	-6.729751	-0.946304	0.503580
C	-7.679256	0.206888	0.112641
C	-7.114735	1.133344	-0.968123
H	1.775895	-0.540145	-1.168939
H	1.820192	-1.054812	2.457474
H	7.986391	-0.077138	-2.346861
H	5.807982	-2.321488	0.610376
H	4.113772	1.260890	-1.060754
H	6.121576	1.558640	-2.464084
H	7.834473	-2.026306	-0.808445
H	4.247361	1.014670	2.675883
H	3.647961	2.691576	2.553177
H	2.591119	1.421979	3.225467
H	1.781834	2.218641	-0.630985
H	-2.462790	3.499427	-0.493286
H	-3.618352	2.421728	-1.305432
H	-3.547705	2.484374	0.461931
H	-3.828533	0.018167	-1.892854
H	-4.508025	-2.044727	-0.807629
H	-3.412351	-0.295157	1.488577
H	-2.219313	-2.261156	-2.030401
H	-2.100060	-2.086964	0.669783
H	-5.649060	0.110698	2.091510
H	-7.267609	-1.613310	1.193920
H	-6.500361	-1.543402	-0.386624
H	-8.626230	-0.226233	-0.234470
H	-7.921113	0.792890	1.010427
H	-6.897320	0.578019	-1.889471

H -7.826066 1.927863 -1.220035
H -6.184403 1.608615 -0.637329
SCF Energy (B3LYP/6-31G*)= -1549.19872544

2_c0028
B3LYP/6-31G* Geometry
O -0.696234 -0.057028 -1.065763
C 0.193304 0.456104 -0.071744
C -0.375918 1.852043 0.273273
C -1.679269 1.895380 -0.360801
C -1.798727 0.746462 -1.082245
C 0.118076 -0.523213 1.112007
C 1.685085 0.447900 -0.476200
C 2.428593 -0.025954 0.843776
N 1.372440 -0.643816 1.618691
O -0.882712 -1.118539 1.481667
C 3.563394 -1.092891 0.672761
O 3.413229 -2.166634 1.243769
C 4.799876 -0.799773 -0.099393
C 7.189125 -0.414722 -1.509050
C 5.771910 -1.817911 -0.150377
C 5.040164 0.415520 -0.763234
C 6.230929 0.598740 -1.466067
C 6.957135 -1.626471 -0.849114
O 2.952613 1.149732 1.426963
C 3.379871 1.048071 2.782670
O 2.148458 1.667034 -0.973318
O 0.212472 2.694801 0.939168
C -2.637673 3.038227 -0.215354
C -2.973932 0.140533 -1.796833
C -3.751396 -0.830665 -0.837161
C -4.240931 -0.196654 0.437202
O -2.574280 -0.581233 -2.941596
O -2.933844 -1.971623 -0.593844
C -5.486702 0.214795 0.706199
C -6.707997 0.157267 -0.173498
C -7.896224 -0.564119 0.497551
C -7.651127 -2.057464 0.733643
H 1.823009 -0.300061 -1.262902
H 1.600968 -1.435889 2.211318
H 8.115674 -0.263655 -2.056748
H 5.571499 -2.750384 0.366277
H 4.298955 1.204520 -0.745308
H 6.408710 1.538534 -1.981073
H 7.700745 -2.417757 -0.883068
H 4.194726 0.320984 2.900992
H 3.742463 2.040720 3.054232

H 2.547187 0.774926 3.440746
H 2.049160 2.309614 -0.243314
H -3.179198 3.240328 -1.145659
H -3.381465 2.847391 0.567102
H -2.082138 3.938518 0.064870
H -3.651438 0.938361 -2.118026
H -4.585474 -1.199196 -1.441406
H -3.476268 -0.077170 1.204130
H -2.253798 -1.434289 -2.590847
H -2.279213 -1.743470 0.097774
H -5.664536 0.646515 1.693372
H -6.484200 -0.324705 -1.132880
H -7.016800 1.186454 -0.411749
H -8.120624 -0.070185 1.453581
H -8.785771 -0.430883 -0.131830
H -6.774120 -2.221810 1.369297
H -8.513735 -2.527636 1.219157
H -7.474413 -2.582383 -0.213303
SCF Energy (B3LYP/6-31G*)= -1549.19911056

2_c0033
B3LYP/6-31G* Geometry
O -0.695422 -0.644678 -0.721105
C 0.189466 0.276086 -0.078991
C -0.493204 1.652847 -0.251245
C -1.824850 1.355407 -0.741434
C -1.869441 0.010398 -0.951530
C 0.268101 -0.165955 1.392016
C 1.652725 0.213806 -0.572312
C 2.503929 0.344912 0.760835
N 1.551533 0.010765 1.800241
O -0.650495 -0.640202 2.043054
C 3.721904 -0.627918 0.918040
O 3.707552 -1.393791 1.874476
C 4.873830 -0.592491 -0.021271
C 7.117842 -0.661899 -1.695785
C 4.972755 0.294612 -1.107019
C 5.914127 -1.512344 0.215138
C 7.026643 -1.546981 -0.615947
C 6.092102 0.252515 -1.938070
O 2.945999 1.686488 0.800169
C 3.456233 2.148091 2.048303
O 1.970762 1.170268 -1.537879
O 0.043925 2.726719 -0.009733
C -2.881295 2.394982 -0.969061
C -3.016836 -0.904760 -1.276878
C -3.644484 -1.481831 0.042937
C -4.126783 -0.442469 1.019497

O -2.610601 -1.983347 -2.091180
O -2.706630 -2.379660 0.630027
C -5.394370 -0.074316 1.246890
C -6.649334 -0.572268 0.581622
C -7.381568 0.529607 -0.210963
C -8.702611 0.047267 -0.817487
H 1.822578 -0.770583 -1.018959
H 1.884312 -0.469593 2.630606
H 7.987690 -0.687948 -2.346935
H 4.177328 0.999958 -1.311464
H 5.823572 -2.190669 1.056761
H 7.823108 -2.261199 -0.426771
H 6.160685 0.938069 -2.777922
H 2.690006 2.101940 2.830488
H 4.338914 1.576456 2.365483
H 3.741724 3.188528 1.885257
H 1.840626 2.039012 -1.108935
H -2.422323 3.386192 -0.906251
H -3.349741 2.295451 -1.954920
H -3.675381 2.339243 -0.215978
H -3.783782 -0.341418 -1.818278
H -4.467896 -2.113866 -0.301984
H -3.336320 0.031521 1.600541
H -2.190537 -2.610622 -1.471865
H -2.041287 -1.855726 1.121631
H -5.558952 0.693542 2.005082
H -7.331451 -0.952330 1.357501
H -6.439696 -1.416970 -0.084888
H -7.569983 1.386097 0.451033
H -6.719147 0.898578 -1.005467
H -9.197856 0.847045 -1.379426
H -8.541217 -0.792413 -1.504511
H -9.395737 -0.291802 -0.037851
SCF Energy (B3LYP/6-31G*)= -1549.19923447

2_c0036
B3LYP/6-31G* Geometry
O -0.652779 -0.434630 -0.900748
C 0.241231 0.388353 -0.148039
C -0.358226 1.809731 -0.255963
C -1.673462 1.621964 -0.836895
C -1.775763 0.299728 -1.146622
C 0.214616 -0.160498 1.288404
C 1.724179 0.278998 -0.569100
C 2.507272 0.259311 0.811754
N 1.481460 -0.090027 1.773558
O -0.763325 -0.624518 1.854994

C	3.655451	-0.796730	0.952895
O	3.543401	-1.631309	1.843043
C	4.858618	-0.760849	0.080071
C	7.187825	-0.839505	-1.473714
C	5.833437	-1.753147	0.302482
C	5.066390	0.193841	-0.930465
C	6.227509	0.146760	-1.701811
C	6.988296	-1.792079	-0.468458
O	3.022959	1.564291	0.976055
C	3.506354	1.894643	2.275262
O	2.146749	1.285441	-1.438830
O	0.220211	2.832737	0.088653
C	-2.658953	2.732121	-1.043808
C	-2.947337	-0.526031	-1.599020
C	-3.680298	-1.154690	-0.359996
C	-4.165963	-0.156993	0.657732
O	-2.549938	-1.567470	-2.464432
O	-2.828182	-2.141139	0.214066
C	-5.414661	0.305560	0.798975
C	-6.640403	-0.050350	0.000897
C	-7.788143	-0.574022	0.886301
C	-9.063340	-0.871571	0.092087
H	1.865166	-0.677039	-1.082424
H	1.739861	-0.648545	2.581124
H	8.090848	-0.868995	-2.077840
H	5.659013	-2.482841	1.085916
H	4.323247	0.956866	-1.124818
H	6.380111	0.885015	-2.483945
H	7.733673	-2.562105	-0.290295
H	4.348924	1.255520	2.572116
H	3.846203	2.929529	2.210966
H	2.709896	1.822105	3.024643
H	2.039919	2.125448	-0.950336
H	-3.410757	2.761056	-0.246681
H	-2.126884	3.688349	-1.037920
H	-3.189661	2.637161	-1.997373
H	-3.651726	0.114012	-2.140158
H	-4.514642	-1.714051	-0.793444
H	-3.396372	0.204467	1.339012
H	-2.199327	-2.257637	-1.869384
H	-2.165917	-1.690110	0.776985
H	-5.591403	1.022482	1.603052
H	-6.410247	-0.791965	-0.773399
H	-6.993410	0.846822	-0.531802
H	-7.451945	-1.480195	1.406570
H	-8.004639	0.166103	1.669174
H	-9.860337	-1.244630	0.744961
H	-9.437480	0.029023	-0.410393

H -8.882894 -1.630136 -0.679425
SCF Energy (B3LYP/6-31G*)= -1549.19953177

2_c0043
B3LYP/6-31G* Geometry
O -0.694039 -0.758055 -0.751195
C 0.148282 0.209363 -0.120981
C -0.573995 1.557996 -0.348167
C -1.885760 1.202822 -0.853931
C -1.883406 -0.148388 -1.024101
C 0.212315 -0.184313 1.364444
C 1.622246 0.178513 -0.584626
C 2.442082 0.381040 0.759143
N 1.480951 0.047854 1.790804
O -0.702961 -0.668311 2.013013
C 3.688814 -0.544056 0.971705
O 3.678837 -1.280734 1.950831
C 4.860895 -0.496073 0.058126
C 7.147025 -0.534609 -1.559628
C 5.933744 -1.358717 0.356706
C 4.948447 0.349346 -1.061342
C 6.088804 0.322429 -1.863996
C 7.067334 -1.377803 -0.445922
O 2.836751 1.737850 0.762141
C 3.313106 2.255670 2.001525
O 1.929051 1.113124 -1.574937
O -0.076356 2.655513 -0.130371
C -2.969306 2.200754 -1.133331
C -2.993438 -1.110152 -1.344481
C -3.629667 -1.668126 -0.020667
C -4.171584 -0.618374 0.913242
O -2.534622 -2.198475 -2.116858
O -2.674761 -2.512935 0.614779
C -5.454657 -0.272024 1.084616
C -6.666528 -0.819380 0.376932
C -7.354460 0.194218 -0.566568
C -7.931201 1.425659 0.140205
H 1.832135 -0.813875 -0.995019
H 1.812951 -0.395403 2.641838
H 8.033197 -0.548641 -2.188765
H 5.851446 -2.005620 1.223532
H 4.128712 1.009837 -1.314191
H 6.148404 0.975015 -2.730388
H 7.889036 -2.047576 -0.208322
H 3.559286 3.301449 1.810932
H 2.540032 2.202019 2.776489
H 4.213755 1.728881 2.344658

H 1.771262 1.990531 -1.173955
H -3.444535 2.029800 -2.105897
H -3.754795 2.172165 -0.369674
H -2.535086 3.205077 -1.135018
H -3.766433 -0.589346 -1.918805
H -4.421892 -2.339873 -0.363959
H -3.413902 -0.115768 1.513796
H -2.105626 -2.791472 -1.470564
H -2.037604 -1.950573 1.101355
H -5.659384 0.501851 1.824733
H -7.399575 -1.138738 1.133091
H -6.406499 -1.714207 -0.199102
H -6.635516 0.512565 -1.333424
H -8.162168 -0.327352 -1.096892
H -8.451115 2.077415 -0.570872
H -8.653229 1.135221 0.913785
H -7.149265 2.023046 0.622365
SCF Energy (B3LYP/6-31G*)= -1549.19786495

2_c0045
B3LYP/6-31G* Geometry
O -0.639519 -0.546262 -0.967291
C 0.211111 0.328063 -0.222079
C -0.417368 1.728978 -0.406964
C -1.708162 1.485903 -1.021328
C -1.770442 0.150124 -1.278853
C 0.153047 -0.163662 1.233996
C 1.708851 0.238628 -0.593400
C 2.447994 0.297773 0.810172
N 1.402471 -0.043291 1.753281
O -0.830805 -0.628250 1.789877
C 3.620963 -0.717072 1.029550
O 3.498669 -1.526268 1.941486
C 4.856959 -0.670711 0.204108
C 7.250898 -0.722948 -1.248955
C 5.857913 -1.613917 0.508671
C 5.070910 0.247899 -0.838122
C 6.264427 0.213872 -1.559129
C 7.045127 -1.639569 -0.212069
O 2.919548 1.623678 0.936153
C 3.349647 2.021042 2.235272
O 2.134309 1.217673 -1.492393
O 0.125451 2.779168 -0.087384
C -2.709084 2.563770 -1.306523
C -2.907795 -0.722174 -1.731379
C -3.666872 -1.312438 -0.489211
C -4.206631 -0.280077 0.465053
O -2.459259 -1.790919 -2.536267

O -2.812915 -2.252146 0.155693
C -5.467032 0.166605 0.543803
C -6.653979 -0.251275 -0.283882
C -7.821938 -0.827344 0.549396
C -8.479356 0.178281 1.500985
H 1.888405 -0.734595 -1.060290
H 1.649574 -0.562358 2.590101
H 8.179192 -0.742002 -1.813945
H 5.677776 -2.316339 1.315367
H 4.308191 0.971779 -1.095683
H 6.421918 0.923574 -2.366324
H 7.810877 -2.371278 0.029766
H 3.660780 3.062473 2.139256
H 2.531263 1.954176 2.961184
H 4.200371 1.420537 2.584768
H 1.994510 2.074358 -1.042738
H -3.281034 2.360786 -2.217942
H -3.424695 2.681023 -0.484297
H -2.186277 3.517393 -1.430080
H -3.608511 -0.123375 -2.322109
H -4.473640 -1.910546 -0.922909
H -3.466895 0.127264 1.153685
H -2.115315 -2.446775 -1.899925
H -2.178143 -1.761615 0.717263
H -5.674246 0.920655 1.303281
H -6.359528 -0.988772 -1.038689
H -7.025459 0.623729 -0.840187
H -8.580125 -1.208443 -0.147454
H -7.460378 -1.693706 1.118263
H -7.782874 0.532380 2.269078
H -8.849547 1.055683 0.955603
H -9.332446 -0.274857 2.018326
SCF Energy (B3LYP/6-31G*)= -1549.19795818
